# Enhancement of Auranofin-Induced Apoptosis in MCF-7 Human Breast Cells by Selenocystine, a Synergistic Inhibitor of Thioredoxin Reductase

**DOI:** 10.1371/journal.pone.0053945

**Published:** 2013-01-14

**Authors:** Chaoran Liu, Zhong Liu, Meng Li, Xiaoling Li, Yum-Shing Wong, Sai-Ming Ngai, Wenjie Zheng, Yibo Zhang, Tianfeng Chen

**Affiliations:** 1 Department of Chemistry, Jinan University, Guangzhou, China; 2 Guangzhou Jinan Biomedicine Research and Development Center, Guangdong Provincial Key Laboratory of Bioengineering Medicine, National Engineering Research Center of Genetic Medicine, Jinan University, Guangzhou, China; 3 School of Life Sciences, The Chinese University of Hong Kong, Shatin, Hong Kong SAR, China; University of Medicine and Dentistry of New Jersey, United States of America

## Abstract

Thioredoxin system plays an important role in regulation of intracellular redox balance and various signaling pathways. Thioredoxin reductase (TrxR) is overexpressed in many cancer cells and has been identified as a potential target of anticancer drugs. Auranofin (AF) is potent TrxR inhibitor with novel *in vitro* and *in vivo* anticancer activities. Selenocystine (SeC) is a nutritionally available selenoamino acid with selective anticancer effects through induction of apoptosis. In the present study, we demonstrated the synergistic effects and the underlying molecular mechanisms of SeC in combination with AF on MCF-7 human breast cancer cells. The results showed that SeC and AF synergistically inhibited the cancer cell growth through induction of ROS-dependent apoptosis with the involvement of mitochondrial dysfunction. DNA damage-mediated p53 phosphorylation and down-regulation of phosphorylated AKT and ERK also contributed to cell apoptosis. Moreover, we demonstrated the important role of TrxR activity in the synergistic action of SeC and AF. Taken together, our results suggest the strategy to use SeC and AF in combination could be a highly efficient way to achieve anticancer synergism by targeting TrxR.

## Introduction

Thioredoxin (Trx) system plays an important role in regulation of intracellular redox balance and various signaling pathways. The major ubiquitous disulfide reductase responsible for maintaining proteins in their reduced state is thioredoxin, which is reduced by electrons from NADPH via thioredoxin reductase [1].In mammals, both Trx and thioredoxin reductase (TrxR) are expressed as dedicated isoforms for either predominantly cytosolic (Trx1 and TrxR1) or mitochondrial (Trx2 and TrxR2) localization. Knockout mice lacking either of these four genes die early during embryogenesis. A third form of TrxR (TrxR 3)in mammals is also expressed, predominantly in testis [2]. TrxR 1 and 2 were mentioned as important antioxidant selenoproteins as well as glutathione peroxidase (Gpx) [3]. TrxR is overexpressed in many cancer cells and has been identified as a potential target of anticancer drugs. Studies have found that TrxR exhibited protective effects against various cellular stresses, including the growth inhibition, and cell death induced by hydrogen peroxide, tumor necrosis factor-α and chemotherapeutic agents [4], [5], [6]. For instance, cisplatin-resistant human bladder cancer cells and PC-3 prostatic cancer cells displayed increased expression levels of TrxR [6], [7], [8]. Thioredoxin reductase is a homodimetric protein essential for reduction and activation of Trx, each subunit of which has a redox active disulfide/dithiol and a tightly bound flavin adenine dinucleotide group that could mediate the transfer of reducing equivalents from NADPH to a disulfide bond of the substrates [9]. The inhibition of both cytosolic and mitochondrial TrxR can affect the intracellular redox balance and hence alter the mitochondrial membrane permeability and consequent release of the segregated proapoptotic factors, finally resulting in apoptosis of cancer cells [10]. Therefore, TrxR has been identified as a potential target for anticancer drug design.

Auranofin (AF) is a metal phosphine complex that has been introduced into clinical practice of chrysotherapy, a treatment of rheumatoid arthritis with gold-based drugs, following the pioneering studies conducted with gold(I) thiolate compounds [11]. Studies have showed that AF acted as a potent inhibitor of thioredoxin reductase, which could cause the alteration of intracellular redox status, thus resulted in overproduction of reactive oxygen species (ROS) and apoptotic cell death [12]. Interestingly, AF was also found markedly effective against various types of drug-resistant cancer cells, such as human ovarian cancer cells [10]. Taken together, these results support the application potential of AF in cancer chemotherapy.

Selenium (Se) is an essential micronutrient of fundamental importance to humans and animals. In the past decades, Se has been extensively studied as a cancer chemopreventive agent. Several cancer chemoprevention trials have showed that that supplementation of Se at supranutritional levels might be a safe and effective way to prevent cancers [13], [14]. Usually, Se acts as a regulator of intracellular ROS production and thiol redox balance [15]. Se also displayed tumor-selective activities though pro-oxidant effects at supra-nutritional concentrations [12]. Recently, TrxR has been found involved in the anticancer action of Se [13], [14]. For instance, knockdown of TrxR 1 in human lung cancer cells enhanced the cytotoxicity of Se, with the involvement of mitochondrial dysfunction. This study also highlighted that the apoptosis-inducing ability of Se was closely related to TrxR activity [16], [17]. Till now, many studies have showed that, organic selenocompounds, especially natural ones, were highly effective chemopreventive agents with well-documented benefits in reducing mortality rates and lower side effects and genotoxic action, by comparing with inorganic selenocompounds [14], [18], [19]. Selenocystine (SeC), a naturally occurring selenoamino acid, received much attention in the past years due to its application potential in cancer chemotherapy [13], [20]. In our previous works, SeC was identified as a novel agent with broad-spectrum anticancer activities through induction of ROS-mediated p53 activation and mitochondria dysfunction. Despite this potency, SeC possessed great selectivity between human cancer and normal cells and deserved further evaluation as a chemotherapeutic agent for human cancers, however, little information about the effects of SeC on the intracellular Trx system and its application as a chemosensitizer is available.

In the present study, the synergistic effect of SeC and AF was elucidated on MCF-7 human breast adenocarcinoma cells, a TrxR overexpressed cell line. The results showed that the combination of SeC and AF caused significantly higher cell growth inhibition than SeC and AF alone, through induction of apoptosis. Treatment of MCF-7 cells with SeC in combination with AF resulted in increase of subG1 cell population, cleavage of PARP, and activation of caspase-7 and caspase-9. Depletion of mitochondrial membrane potential, alteration in expression level of Bcl-2 family proteins and increase of mitochondria mass, also occured in cells exposed to SeC and AF. Moreover, SeC and AF effectively down-regulated the phosphorylation of ERK and AKT, and induced the decrease of MDM2 phosphorylation and increase of p53 phosphorylation. Furthermore, the roles of intracellular TrxR activity, TrxR1 and Trx protein expression levels in cell apoptosis, were also investigated, and the results showed that the cellular redox status, as well as the TrxR1 activity rather than its expression level, were closely related to the anticancer of SeC and AF in combination. Taken together, our results suggest that the combination of SeC and AF could be a novel strategy to achieve anticancer synergy by targeting TrxR system.

## Materials and Methods

### 1. Cell Culture

The human breast adenocarcinoma MCF-7 cell line was obtained from American Type Culture Collection (ATCC, Manassas, VA) and maintained in DMEM medium supplemented with fetal bovine serum (10%, containing 50.5 ng/mL Se), penicillin (100 units/ml) and streptomycin (50 units/ml) at 37°C in a humidified incubator with 5% CO_2_ atmosphere.

### 2. Drug Treatment

MCF-7 cells were seeded in 6-well tissue culture plates at 4×10^4^ cells/well for 24 h. For the co-treatment, the cells were incubated with different concentrations of SeC for 24 h followed by simultaneously treatment with SeC and AF (4 µM) for 6 h. For the SeC treatment, the cells were incubated with different concentrations of SeC for 30 h. For the AF treatment, the cells were incubated with AF (4 µM) for 6 h, with or without pretreatment of 20 µM U0126 or LY294002 for 1 h prior to the addition of agents. For the experiments regarding the N-acetyl cysteine (NAC) protection, the cells were pretreated with 5 mM NAC for 2 h prior to the addition of other agents.

### 3. MTT Assay

The cell viability was determined by MTT assay as previously described [18]. Briefly, the cells were seeded in 96-well tissue culture plates at 4×10^4^ cells/well for 24 h, and then incubated with SeC or AF alone or in combination as described in drug treatment. After incubation, 20 µL/well of MTT solution (5 mg/mL in PBS) was added and incubated for 5 h. The medium was aspirated and replaced with 150 µL/well of DMSO to dissolve the formazan salt formed. The color intensity of the formazan solution, which reflects the cell growth condition, was measured at 570 nm using a microplate spectrophotometer (VersaMax). The cell viability of treatment groups was expressed as percentage of the control.

### 4. Flow Cytometric Analysis

Cell cycle distribution was monitored by flow cytometric analysis. Briefly, cells after treatments were trypsinized, washed with PBS and fixed with 75% ethanol overnight at −20°C. The fixed cells were washed with PBS and stained with propidium iodide (PI) working solutions (1.21 mg/ml Tris, 700 U/ml RNase, 50.1 mg/ml PI, pH 8.0) for 4 h in darkness. The stained cells were then subjected to an Epics XL-MCL flow cytometer (Beckman Coulter, Miami, FL). Cell cycle distribution was analyzed using MultiCycle software (Phoenix Flow Systems, San Diego, CA). The proportion of cells in G0/G1, S, G2/M phases was represented as DNA histograms. Apoptotic cells with hypodiploid DNA content were measured by quantifying the sub-G1 peak in the cell cycle pattern. For each experiment 10000 events per sample were recorded.

### 5. TUNEL and DAPI Co-staining Assay

Cells cultured in chamber slides were fixed with 3.7% formaldehyde for 10 min and permeabilized with 0.1% Triton X-100 in PBS. After then, the cells were incubated with 100 µL/well TUNEL reaction mixture containing nucleotide mixture and terminal deoxynucleotidyl transferase (TdT) for 1 h and 1 µg/ml of DAPI for15 min at 37°C, respectively. The cells were then washed with PBS and examined under a fluorescence microscope (Nikon Eclipse 80i). The apoptotic percentages, as calculated by dividing the TUNEL-positive cell number by the total cell number (DAPI-positive) within the same area, are listed in the parentheses. All results were obtained from three independent experiments. Significant difference between treatment and control groups is indicated at *P*<0.05 (*) or *P*<0.01 (**) level.

### 6. Evaluation of Mitochondrial Membrane Potential (ΔΨm)

Cells cultured in six-well plates were trypsinized and resuspended in 0.5 ml of PBS buffer containing 10 mg/ml JC-1. After incubation for 10 min at 37°C in the incubator, cells were immediately centrifuged to remove the supernatant. Cell pellets were suspended in PBS and then analyzed by flow cytometry. The percentage of the green fluorescence from JC-1 monomers was used to represent the cells that lost ΔΨm.

### 7. Measurement of Mitochondrial Mass

The mitochondrial mass was determined using the fluorescent dye NAO. Briefly, the treated cells were trypsinized and resuspended in PBS buffer containing 10 µM of NAO for 15 min at 37°C in the dark. The staining cells were washed with PBS, and the number of viable cells was counted with a hemacytometer. The intensity of NAO in staining cells was then analyzed by flow cytometer.

### 8. Measurement of ROS Generation

Cells were harvested, washed, and suspended in PBS (1×10^6^ cells/ml) containing 10 µM of DCFH-DA. After incubation at 37°C for 30 min, the cell were collected and resuspended in PBS. The ROS level was then determined by measuring the fluorescence intensity on a Tecan SAFIRE multifunctional monochromator based microplate reader, with the excitation and emission wavelengths set at 488 and 525 nm, respectively.

### 9. Determination of TrxR Activity

Cytosolic extracts were prepared by incubating the cells on ice in hypotonic buffer (20 mM Hepes, 10 mM KCl, 1.5 mM MgCl_2_, 1 mM EDTA, 1 mM EGTA, 250 mM sucrose, 1 mM dithiothreitol, aprotinin, leupeptin and pepstatin 2 mg/ml each, pH 7.5) for 30 min. Then the cells were disrupted in a Dounce homogenizer by optimal gentle strokes and centrifuged at 1000 g for 10 min at 4°C to remove unbroken cells and nuclei. The homogenates were collected and centrifuged again at 12 000 g for 30 min at 4°C to separate the mitochondria and cytosol fraction. The protein concentrations of the supernatants were determined using the Bio-Rad assay kit. TrxR activity was determined by end point insulin reduction assay modified from Arner et al [21] with slight modifications. Briefly, 50 µg of the cytosolic extract were incubated with 2.5 mg/ml bovine insulin, 2 mM EDTA, 400 µM NADPH, 0.8 µM Trx1 in 60 µl of 100 mM HEPES (pH 7.4) at 37°C for 30 min. The reaction was terminated by addition of 200 µl of 6 M guanidine hydrochloride/1 mM DTNB. The reaction mixtures with the omission of Trx1 were used as the control. The percentage of TrxR1 activity in comparison with the control was determined by measuring the absorbance at 412 nm. TrxR2 activity of the cell mitochondria extract was determined by TrxR assay Kit (Item No. 10007892) purchased from Cayman Chemical Company.

### 10. Determination of Glutathione Peroxidase (GPx) Activity

GPx activity was measured by quantifying the rate of oxidaion of reduced glutathione to oxidized glutathione by the hydrogen peroxide. One unit of GPx activity was defined as the decrease in 1 mM GSH (except the effect of nonenzymatic reaction) in system of enzymatic reaction of 1 mg of protein/min. Determination of GPx activity was based on the oxidation of GSH by GPx, coupled to the disappearance of NADPH by GR. The decrease in absorbance at 340 nm was measured.

### 11. Western Blot Analysis

Total cellular proteins were extracted by incubating cells in lysis buffer obtained from Cell Signaling Technology. The protein concentrations in the cell lysates were determined by bicinchoninic acid assay (Sigma) according to the manufacturer’s instructions. SDS-PAGE was done in 10% tricine gels loading equal amount of proteins per lane. After electrophoresis, separated proteins were transferred to nitrocellulose membrane and blocked with 5% non-fat milk in Tris-Buffered-Saline with Tween (TBST) for 1 h. After that, the membranes were incubated with primary antibodies at 1∶1000 dilutions in 5% non-fat milk overnight at 4°C, and then secondary antibodies conjugated with horseradish peroxidase at 1∶2000 dilution for 1 h at room temperature. Protein bands were visualized on X-ray film using an enhanced chemiluminescence system (Kodak). To assess the presence of a comparable amount of proteins in each lane, the membranes were stripped finally to detect the β-actin. The protein expression levels were calculated by Quantity One basic 4.6.3 software (Bio-Rad Laboratories, Hercules, CA, USA).

### 12. Statistical Analysis

Experiments were carried out at least in triplicate and results were expressed as mean ± SD. Statistical analysis was performed using SPSS statistical package (SPSS 13.0 for Windows; SPSS, Inc. Chicago, IL). Difference between two groups was analyzed by two-tailed Student’s t test, and that between three or more groups was analyzed by oneway ANOVA multiple comparisons. Difference with *P*<0.05 (*) or *P*<0.01 (**) was considered statistically significant. The synergistic effect between SeC and AF was evaluated by the isobologram method [22]. Briefly, a straight line was formed by plotting the IC50 values of SeC and AF on the x- and y-axes, respectively. The data point in the isobologram corresponds to the actual IC50 value of combination of SeC with AF.If a data point is on or near the line, this represents an additive treatment effect, whereas a data point that lies below or above the line indicates synergism or antagonism, respectively. Moreover, the combination index (CI) was also calculated to examine the interaction between SeC and AF. A CI value of 1 indicates an additive effect between two drugs. Synergism is reflected by a combination index of <1, whereas antagonism is reflected by a combination index of >1.

## Results

### 1. Synergistic Apoptosis-inducing Effect of SeC and AF on MCF-7 Cells

**Figure 1:**
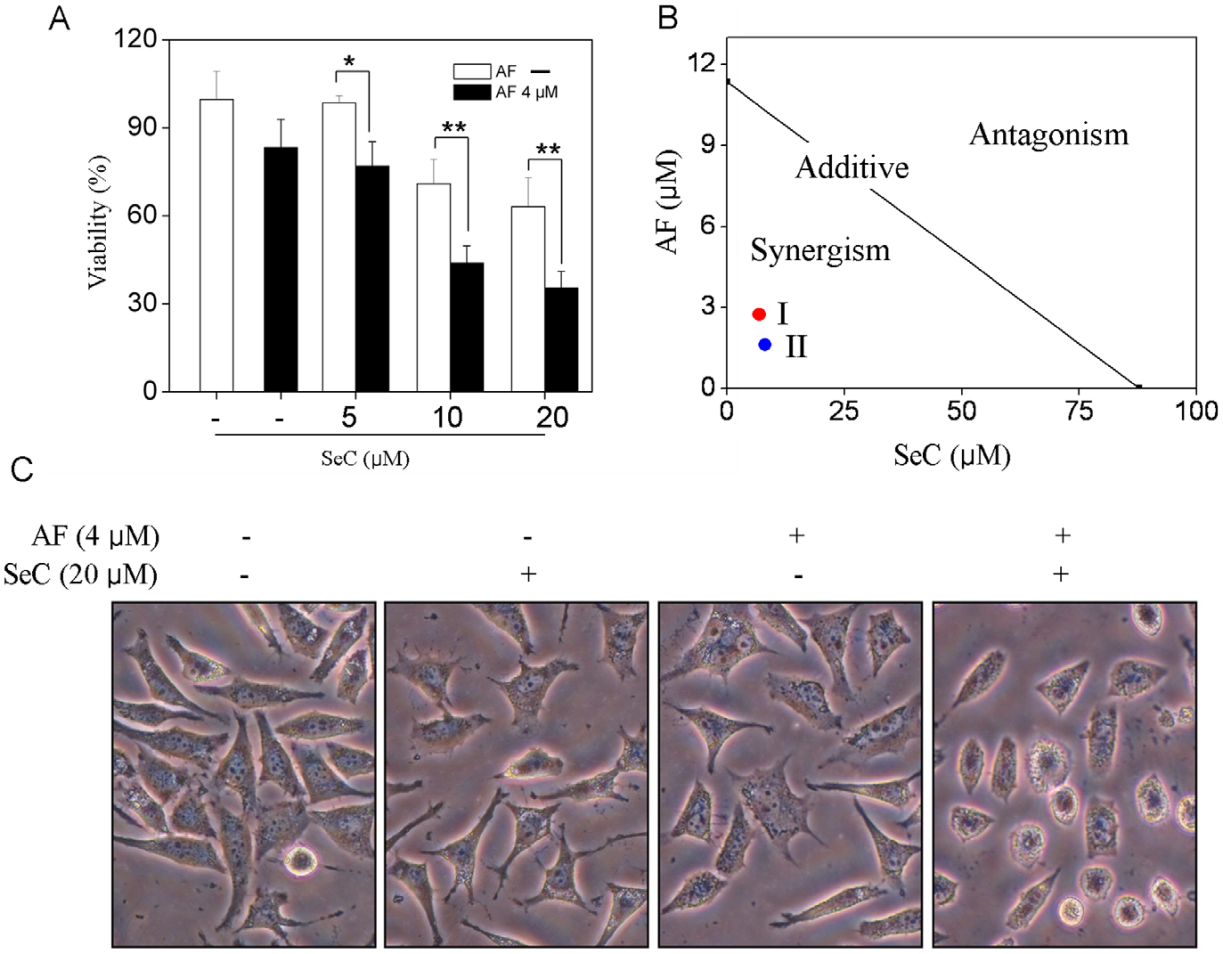
SeC enhances AF-induced cytotoxicity in MCF-7 cells. Cells were pretreated with or without 5–20 µM SeC for 24 h and then cultured in the presence or absence of 4 µM AF for 6 h. (**A**) Cell viability as examined by MTT assay. (**B**) Isobologram analysis of the antiproliferative effects of SeC and AF on MCF-7 cells. (**C**) Morphology of MCF-7 cells under different treatments. All data were obtained from three independent experiments and presented as the means ± SD. Bars with different characters are statistically different at *P<0.05, **P<0.01.

In the present study, the synergistic effect of SeC and AF was examined on MCF-7 human breast adrenocarcinoma cells, a TrxR overexpression cell line. As shown in Fig. 1A, the combination of SeC and AF caused significantly stronger cell growth inhibition than SeC and AF alone, No significant change in the proliferation of MCF-7 cells treated with AF for 6 h was observed. Interestingly, pretreatment of the cells with SeC for 24 h followed by simultaneous incubation with AF for 6 h resulted in much stronger growth inhibition by comparing with either SeC or AF alone. The results of microscopic examination (Fig. 1C) also revealed the occurence of cell shrinkage, cell rounding, and the appearance of apoptotic bodies after treatments.

To determine whether the anticancer action of SeC and AF in MCF cells were synergistic, addictive, or antagonistic, the growth inhibitory effects or different treatments were examined by MTT assay and analyzed by the isobologram method [22]. As shown in Fig. 1B, the *in vitro* anticancer activities of SeC and AF under different ratios (5∶2 and 5∶1) were investigated by comparing with the individual drugs. The IC_50_ values of SeC and AF on MCF-7 cells were found at 87.8 µM and 11.4 µM, respectively, while the IC_50_ values for the combined treatments of SeC and AF under the two tested ratios were both 9.6 µM. The results of the isobologram analysis revealed that the growth inhibitory effects between SeC and AF was strongly synergistic, as evidenced by the location of the data point in the isobologram being far below the line defining an additive effect. Moreover, the actual IC_50_ value of the combination (9.6 µM) was significantly lower than the theoretical ones under different ratios (30.0 µM for 5∶2 and 41.4 µM, 5∶1). The combination index (CI) of the IC_50_ value of the SeC and AF was found at 0.32 (5∶2) and 0.23 (5∶1), which further confirmed the synergism between SeC and AF. Taken together, our results clearly demonstrate that the strategy to combine SeC and AF could be a highly efficient way to enhance its anticancer efficacy. Moreover, SeC in combination with AF at the ratio of 5∶1 was selected as an optimized condition for further studies.

**Figure 2:**
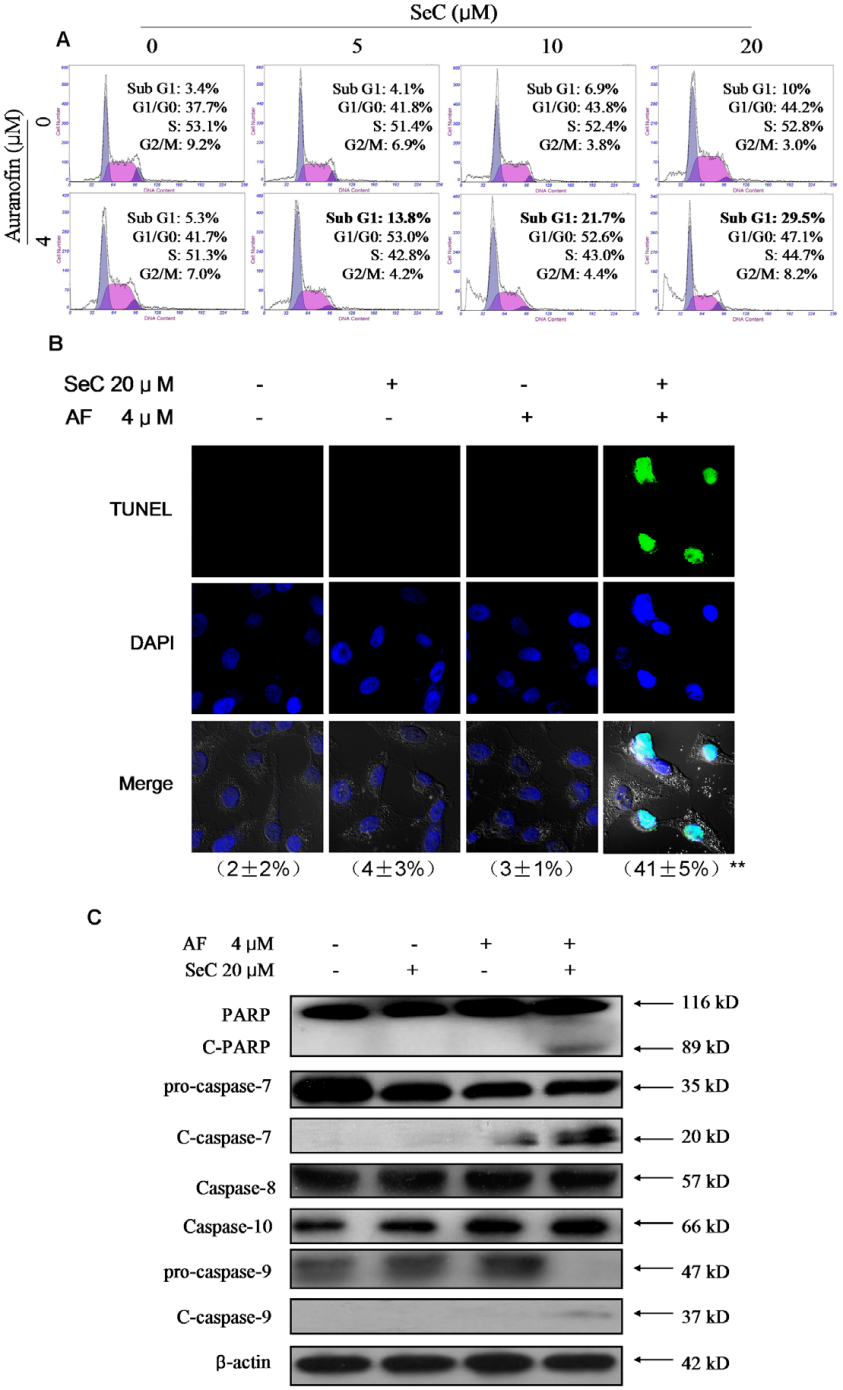
Synergistic induction of apoptosis by SeC and AF in MCF-7 cells. Cells were pretreated with or without 5–20 µM SeC for 24 h and then cultured in the presence or absence of 4 µM AF for 6 h. (**A**) SeC increased AF-induced sub-G1 population accumulation. Quantitative analysis of apoptotic cell death induced by SeC or/and AF by flow cytometric analysis. (**B**) Representative photomicrographs of DNA fragmentation and nuclear condensation in response to SeC or/and AF treatment, as detected by TUNEL assay and DAPI staining. Top: DNA fragmentation was detected by TUNEL assay (magnification, 200×). TUNEL-positive nuclei due to DNA fragmentation appear as green color. Middle: Evaluation of apoptosis in MCF-7 cells was examined by DAPI staining (magnification, 200×) Bottom: Merged images of DAPI staining and TUNEL for the same area. The images shown here are representative of three independent experiments with similar results. The apoptotic percentages, as calculated by dividing the TUNEL-positive cell number by the total cell number (DAPI-positive) within the same area, are listed in the parentheses. All results were obtained from three independent experiments. Significant difference between treatment and control groups is indicated at *P*<0.05 (*) or *P*<0.01 (**) level. (C) Western blot analysis the quantitative of PARP and caspases cleaved in the apoptosis induced by SeC or/and AF. Equal protein loading was confirmed by analysis of β-actin in the protein extracts. Similar results were obtained from three independent experiments.

In order to elucidate the intracellular mechanisms for the synergistic effects of SeC and AF, firstly, we performed a DNA flow cytometric analysis to examine the change in cell cycle distribution. As reflected by the Sub-G1 cell populations (Fig. 2A), no significant apoptosis was observed in cells exposed to AF alone. Treatment of SeC alone resulted in slight increase in the percentage of apoptotic cells from 3.4% (control) to 10.0% (20 µM). However, significant increase in Sub-G1 cell populations was observed in cells co-incubated with SeC and AF. For instance, the cells exposed to SeC (20 µM) and AF (4 µM) displayed 29.5% of apoptosis. To further confirm the induction of apoptosis, we detected the DNA fragmentation and nuclear condensation as apoptotic markers by TUNEL and DAPI co-staining assay. As shown in Fig. 2B, the cells treated with SeC and AF in combination showed significant DNA fragmentation and nuclear condensation (41%), which were not detected in the cells treated with SeC or AF alone (<5%). These results indicate that apoptosis is the major mode of cell death induced by combined treatment of SeC and AF.

This finding was further confirmed by cleavage of PARP and caspases as examined by Western blotting. Caspases, a family of cysteine acid proteases, are known to act as important mediators of apoptosis and contribute to the overall apoptotic morphology by cleavage of various cellular substrates. Caspase-7, like caspase-3, is an effector caspase that is responsible for cleaving downstream substrates such as PARP. Due to the lack of expression of casepase-3 in MCF-7 cells, caspase-7 has been identified as a major contributor to the execution of apoptosis in this cell line [23]. As shown in Fig. 2C, expose of MCF-7 cells to combined treatment of SeC and AF resulted in cleavage of caspase-7, which subsequently induced the proteolytic cleavage of PARP, a protein serving as a biochemical hallmark of cells undergoing apoptosis. However, SeC or AF alone showed no effects on caspase and PARP activation. Moreover, the activation of two kinds of initiator caspases, caspase-8 and caspase-10 (Fas/TNF-mediated) and caspase-9 (mitochondrial-mediated) was also examined by Western blot analysis. As shown in Fig. 2C, the combined treatment of SeC and AF effectively triggered the cleavage of caspase-9, which was not observed in cells exposed to SeC or AF alone. Furthermore, no change in the expression levels of caspase-8 and caspase-10 was detected in treated cells. These results suggest that, mitochondria play an important role in the regulation of MCF-7 cell apoptosis induced by SeC and AF in combination.

### 2. SeC and AF Synergistically Induce Apoptosis in MCF-7 Cells with the Involvement of Mitochondrial Dysfunction

**Figure 3:**
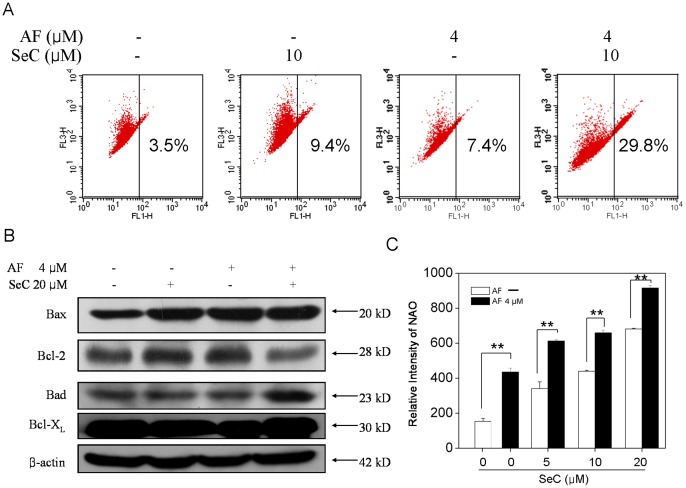
Mitochondrial membrane depolarization and alteration in the expression level of Bcl-2 family proteins triggered by SeC between AF. Cells were pretreated with or without 5–20 µM SeC for 24 h and then cultured in the presence or absence of 4 µM AF for 6 h. (**A**) Treated cells were harvested and stained with the mitochondria-selective dye JC-1 and then analyzed by flow cytometry. The number in the right region of each dot plot represents the percentage of cells that emit green fluorescence due to the depletion of △Ψm. (**B**) Western blot analysis of the expression levels of Bad, Bcl-xl, Bax, Bcl-2 in MCF-7 cells. Equal protein loading was confirmed by analysis of β-actin in the protein extracts. Similar results were obtained from three independent experiments. (**C**) SeC and AF induced mitochondrial mass increase in MCF-7 cells. Mitochondrial mass was determined by flow cytometric analysis after staining the cells with NAO. Results were expressed as percentages of the fluorescence intensity of control cells. Bars with different characters are statistically different at *P<0.05, **P<0.01.

Mitochondria act as a point of integration for apoptotic signals originating from both the extrinsic and intrinsic pathways. Depletion of mitochondrial membrane potential (ΔΨm) is a crucial step in the apoptotic process and is lethal to the cells, because it leads to the release of diverse apoptogenic factors from mitochondria into cytoplasm [24]. Our previous results have demonstrated that the combination of SeC and AF triggered cancer cell apoptosis through mitochondria-mediated pathway. Therefore, we examined the change of ΔΨm in MCF-7 cells by flow cytometric analysis by using JC-1 as a molecular probe. JC-1 is a cationic dye that exhibits potential-dependent accumulation in mitochondria. During the loss of ΔΨm, the fluorescence of JC-1 dye shifts from red to green. Therefore, the increase in green fluorescence indicates the loss of ΔΨm in the treated cells. As shown in Fig. 3A, the combination of SeC and AF led to much higher depletion of ΔΨm (29.8%) than either SeC (9.4%) or AF (7.4%) alone.

Bcl-2 family members have been identified as key regulators of mitochondria permeability [25], [26], [27]. Therefore, we examined the effects of SeC and AF on the expression levels of pro-survival and pro-apoptotic Bcl-2 family proteins in MCF-7 cells by Western blotting. As shown in Fig. 3B, co-treatment of the cells with SeC and AF significantly increased the expression of the pro-apoptosis protein Bad. Interestingly, almost no changes in Bad expression occurred in cells treated with SeC or AF alone. Another pro-apoptosis protein, Bax, increased drastically in all treatments. Moreover, NAO was selected as a marker of mitochondrial function. As shown in Fig. 3C, SeC enhanced the mitochondrial mass increase induced by AF in MCF-7 cells. These results suggest that SeC and AF cooperatively induced apoptosis via the mitochondrial dysfunction.

### 3. Contribution of DNA Damage and p53 Phosphorylation to Apoptosis Induced by SeC and AF

**Figure 4:**
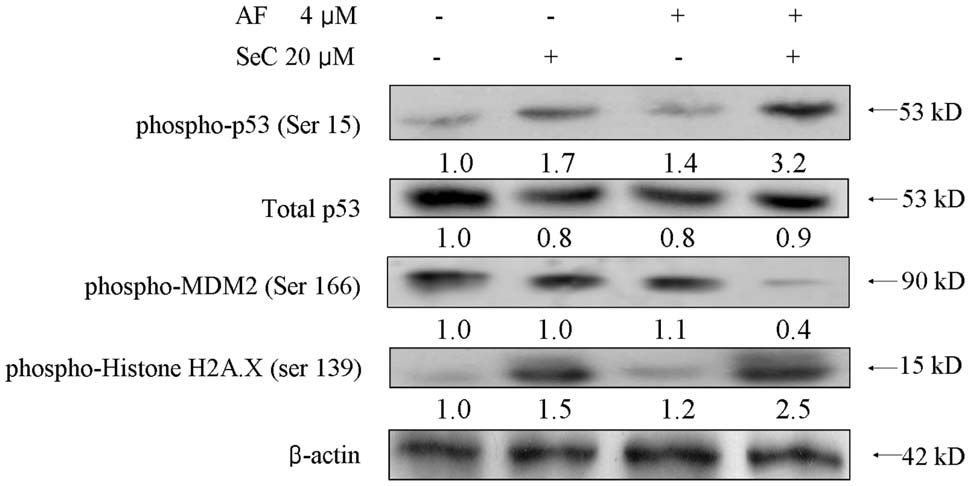
SeC enhances AF-induced apoptosis by triggering p53 phosphorylation. Cell were pretreated with or without 20 µM SeC for 24 h and then cultured in the presence or absence of 4 µM AF for 6 h. The expression levels of phosphorylation of p53, histone H_2_A, MDM2 and total p53 in MCF-7 cells were analyzed by Western blotting. Equal protein loading was confirmed by analysis of β-actin in the protein extracts. Similar results were obtained from three independent experiments.

The above results have showed that treatments of the cells with AF and SeC up-regulated the expression levels of p53-inducible genes (PIGs), including Bax and Bad in MCF-7 cells, which suggest the possible involvement of p53 pathway in apoptosis induced by co-treatment of SeC and AF. Moreover, our previous studies have reported that apoptosis induced by SeC in MCF-7 cells was p53-dependent [14]. Thus, the levels of total p53 and phosphorylated p53 were examined by means of Western Blotting. As shown in Fig. 4, treatment of SeC alone resulted in slight increase of phosphorylated p53 (Ser 15), while AF alone had no effect on the status of p53. However, significant increase in p53 phosphorylation was detected in cells co-incubated with SeC and AF. While no significant changes in total p53 protein expression was observed in all treatments. Ser139-Histone H2A.X, a marker of DNA damage, was also up-regulated in MCF-7 cells treated by SeC and AF, which was consistent with of the change of phosphorylated p53. Moreover, co-treatment with SeC and AF significantly suppressed the phosphorylation of MDM2, a regulator of p53 degradation. Taken together, these results indicate that activation of p53 pathway is required for the synergistic anticancer action of SeC and AF.

### 4. Contribution of AKT Phosphorylation to Cell Apoptosis

**Figure 5:**
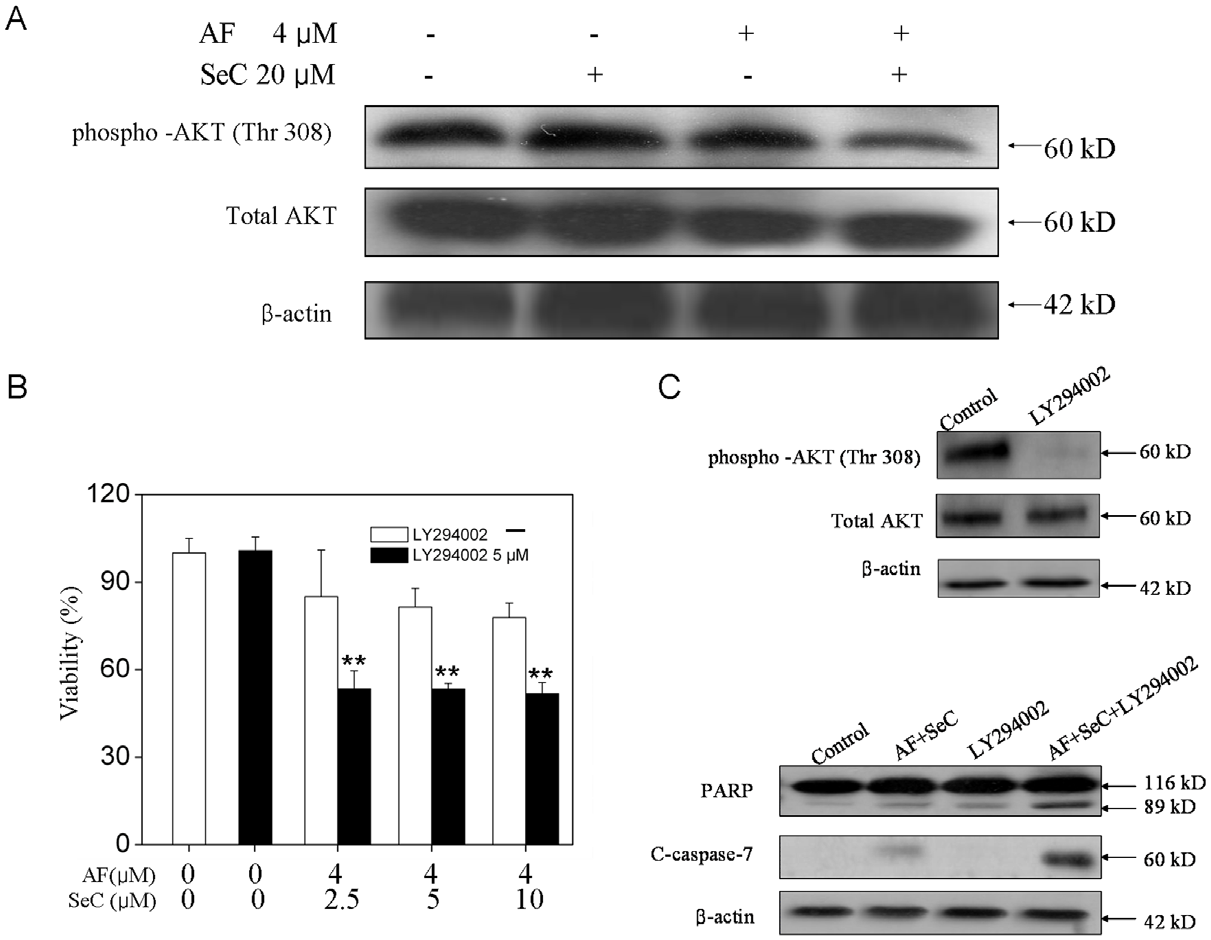
SeC synergizes with AF to induce apoptosis with the involvement of AKT signaling pathway. (**A**) Effects of SeC or/and AF on the phosphorylation and expression level of AKT. Cells were pretreated with or without 20 µM SeC for 24 h and then cultured in the presence or absence of 4 µM AF for 6 h. (**B**) Effect of LY294002 (PI3K inhibitor) on growth inhibition induced by SeC and AF. Cells were pretreated with 10 µM LY294002 for 1 h and then cultured in SeC for 24 h, followed by cultured in the presence of AF for 6 h. Cell viability was determined by the MTT assay, All data are expressed as means ± SD of triplicates. Bars with different characters are statistically different at *P<0.05, **P<0.01. (**C**) cell lysates were subjected to Western blot analysis.PARP cleavage and caspase-7 cleavage were determined. Equal protein loading was confirmed by analysis of β-actin in the protein extracts. Similar results were obtained from three independent experiments.

Several protein kinase pathways have been known to regulate cell proliferation and survival. AKT is the major signal molecule closely related to the activation of p53 in most cell types [28]. The PI3K/AKT pathway is frequently activated in breast cancer cells, which results in enhanced resistance to apoptosis through multiple mechanisms [29]. In the present study, the activation of p53 pathway (Fig. 4) also suggested the possible involvement of AKT in cell apoptosis. In order to investigate the possible role of AKT pathway in the synergistic action of SeC and AF, we examined the expression levels of phospho-AKT in cells treated with SeC and AF by Western blot analysis. As shown in Fig. 5A, the combination of SeC and AF significantly triggered the dephosphorylation of AKT at the site of Ser 308, which could not be observed in cells exposed to SeC or AF alone. In contrast, the expression of total AKT was not affected by all treatments. To further confirm the importance of AKT activation in cell apoptosis, a chemical inhibitor of AKT (LY294002) was employed to block the activity of PI3K, which is an upstream activator of AKT. The presence of the LY294002 was expected to overcome the barrier of cell apoptosis induced by SeC and AF. As shown in Fig. 5B, pretreatment of the cells with LY294002 resulted in a mark decrease in cell viability. The cleavage of PARP and caspase-7 induced by AF and SeC was significantly enhanced by LY294002 (Fig. 5C). Taken together, these results indicate that dephosphorylation of AKT was associated with the apoptosis induced by the synergy of SeC and AF.

### 5. Contribution of MAPKs to Cell Apoptosis

**Figure 6:**
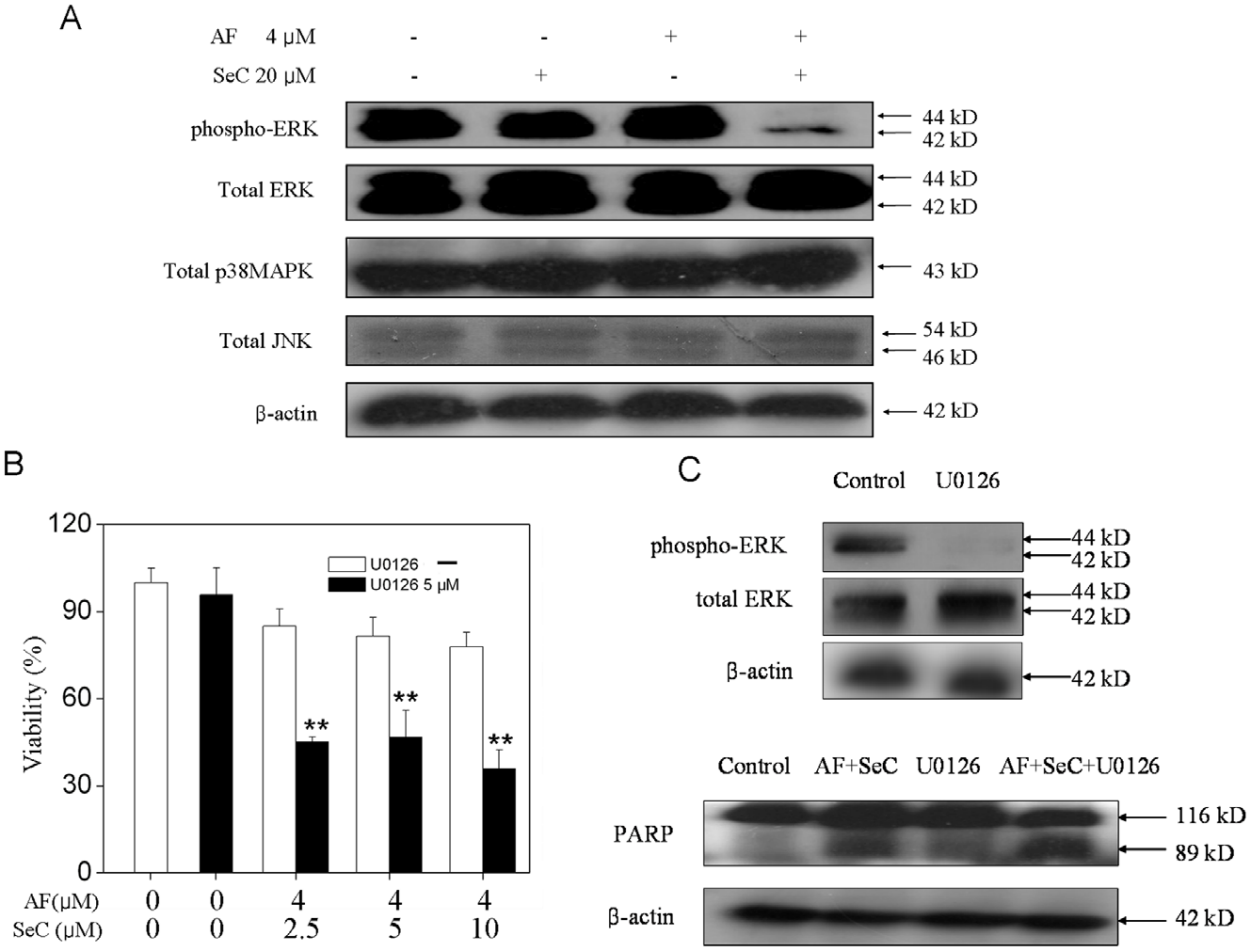
Contribution of MAPKs to apoptosis induced by SeC and AF. (**A**) Effects of SeC or/and AF on the phosphorylation and expression level of ERK, p38MAPK and JNK. (**B**) Effect of U0126 (MEK inhibitor) on growth inhibition induced by SeC and AF. Cells were pretreated with 10 µM U0126 for 1 h and then cultured in SeC (20 µM) for 24 h, followed by cultured in the presence of AF(4 µM) for 6 h. Cell viability was determined by the MTT assay, All data are expressed as means ± SD of triplicates. Bars with different characters are statistically different at *P<0.05, **P<0.01. (**C**) cell lysates were subjected to Western blot analysis. PARP cleavage and caspase-7 cleavage were determined. Equal protein loading was confirmed by analysis of β-actin in the protein extracts. Similar results were obtained from three independent experiments.

Studies have showed that MAPK signaling pathways played important roles in the action of chemotherapeutic drugs [30]. In this study, experiments were conducted to investigate the roles of MAPKs in the synergy between SeC and AF. As shown in Fig. 6A, combined treatment of cells with SeC and AF effectively decrease the phosphor-ERK, which was not observed in cells exposed to SeC or AF alone. Moreover, no alteration in the phosphorylation status of JNK and p38 was detectable in treated cells. To further examine the contribution of ERK inactivation to cell apoptosis, we examined the effects of specific MEK inhibitor (U0126) on the overall cell death induced by the combined treatment. The results of MTT assay showed that pretreatment of the cells with U0126 significantly enhanced SeC and AF-induced cell growth inhibition (Fig. 6B). Consistent with this result, U0126 also effectively enhanced the PARP cleavage induced by SeC and AF (Fig. 6C). Taken together, these results revealed that, inactivation of ERK pathway contributes to the synergistic effects of SeC and AF.

### 6. ROS-dependent Apoptosis Induced by Cooperative Treatment

**Figure 7:**
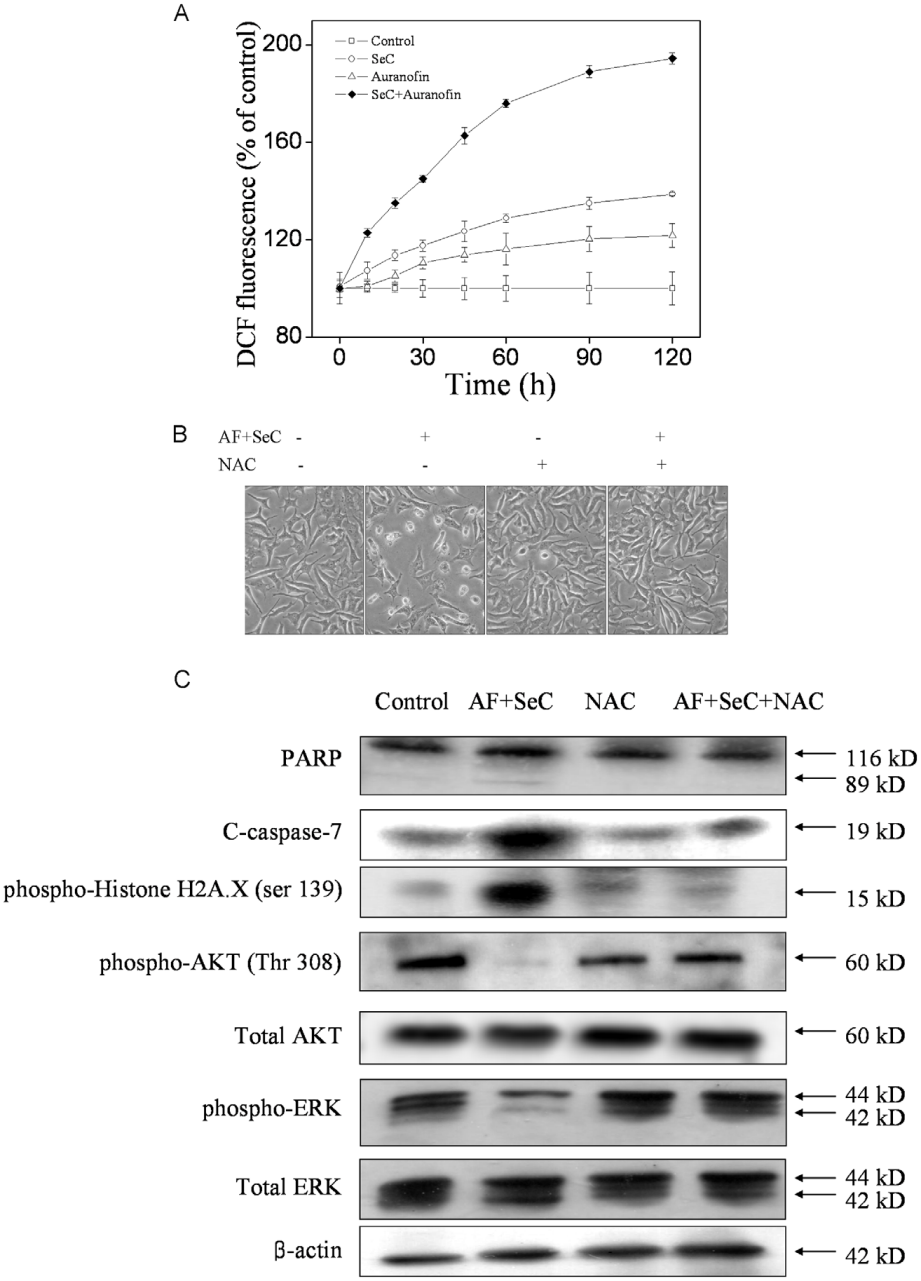
Roles of ROS in apoptosis induced by SeC and AF. (**A**) Effects of SeC or/and AF on ROS generation in MCF-7 cells. MCF-7 cells were washed and treated with SeC (20 µM) and AF (4 µM) after treatment with 10 µM DCFH-DA for 25 min, Fluorescence was analyzed as described in section of Materials and methods. (**B**) Morphology and (**C**) Western blot analysis of MCF-7 cells under treatment with SeC and AF, NAC, or the combination. MCF-7 cells were pretreated with 5 mM NAC for 2 h and further treated with 20 µM SeC for 24 h, and then AF was added for another 6 h-incubation. Cell lysates were subjected to Western blot analysis. Protein level of PARP, cleaved caspase-7, phosphorylated histone H_2_A, phosphorylated AKT, total AKT, phosphorylated ERK, total ERK were determined. Equal protein loading was confirmed by analysis of β-actin in the protein extracts. Similar results were obtained from three independent experiments.

ROS plays an important role in cancer cell apoptosis induced by anticancer drugs that cause DNA damage in the cell nucleus. The status of the cellular redox system are closely relate to many kinds of kinase, including AKT, MAPKs and the downstream effectors [13], [14]. In our previous studies, SeC-induced apoptosis in cancer cells have been found depending on intracellular ROS generation [14]. The main source of ROS inside the cells was the mitochondrial respiratory. In this study, the observation of mitochondrial dysfunction (Fig. 3) suggested that ROS could play an important role in the cell apoptosis induced by SeC and AF. Therefore, the intracellular ROS generation was measured by using a fluorescent probe dicholorofluorescein (DCF). The assay is based on the cellular uptake of a non-fluorescent probe (DCFH-DA), which is subsequently hydrolyzed by intracellular esterases to form dichlorofluorescin (DCFH). This non-fluorescent substrate is oxidized by intracellular ROS producing a fluorescent product DCF. The MCF-7 cells were treated with SeC (20 µM) alone or in combination with AF (4 µM) following incubation with the fluorescent probe DCFH-DA. As shown in Fig. 7A, during the 2-h incubation, only slight increase in intracellular ROS generation was observed in cells exposed to SeC or AF alone. In contrast, the combined treatment induced much higher intracellular ROS generation in MCF-7 cells.

To further verify the role of ROS generation in the cells apoptosis, ROS scavengers were employed to examine whether inhibition of ROS could block the apoptosis induced by the combined treatment. The results of microscopic examination revealed that pretreatment of the cells with NAC (5 mM) completely suppressed the cell death induced by SeC and AF (Fig. 7B). Moreover, NAC also reversed the combined effects of SeC and AF, including the PARP cleavage and caspase-7 cleavage, phosphorylation of p53, Histone H2A.X, and dephosphorylation of AKT and ERK (Fig. 7C). Taken together, these results suggest that the synergistic apoptosis-inducing effects between SeC and AF occur in a ROS-dependent manner.

### 7. SeC and AF Cooperatively Induce Cell Apoptosis by Targeting TrxR

**Figure 8:**
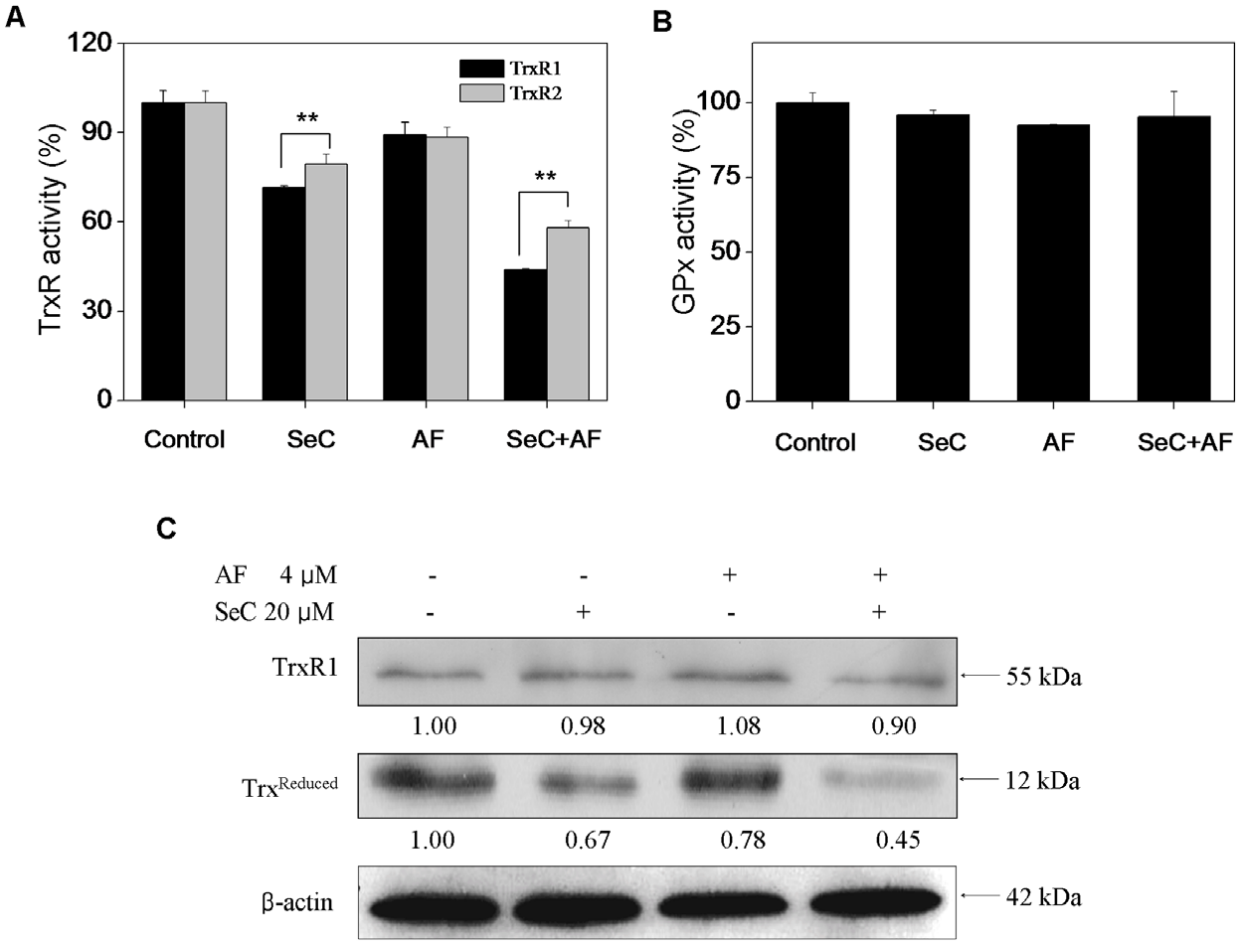
Changes of antioxidant enzyme activities of TrxR and GPx in MCF-7 cells induced by SeC and AF. Enzymatic activity of TrxR1, TrxR2 (**A**) and GPx (**B**) in MCF-7 cells after treatment with SeC or/and AF. Cells were pretreated with or without 20 µM SeC for 24 h and then cultured in the presence or absence of 4 µM AF for 6 h. Bars with different characters are statistically different at *P*<0.01. (**C**) Cell lysates were subjected to Western blot analysis, protein levels of TrxR1 and Redox thioredoxin were examined. Equal protein loading was confirmed by analysis of β-actin in the protein extracts. Similar results were obtained from three independent experiments.

AF has been identified as a potent inhibitor of both mitochondrial and cytosolic TrxR [31]. Similarly, several selenocompounds were also found be able to compete with Trx to inhibit TrxR activity [31]. In the present study, to investigate the combined effects of SeC and AF on TrxR activity in cell lysates, the insulin reduction assay was performed, and the measurement of total GPx activity was also performed. Fig. 8A showed that treatment of the cells with AF or SeC alone resulted in slight decrease in the activities of TrxR1 and TrxR2 to 81.9% and 82.5% (AF, 4 µM), 72.0% and 80.3% (SeC, 20 µM), respectively. In contrast, significant decrease in TrxR1 and TrxR2 activity (41.3% and 58.1%) was observed in cells cooperatively treated with SeC (20 µM) and AF (4 µM). Moreover, we found that, the inhibitory effects of SeC and the combined treatment on TrxR1 were significantly stronger than those on TrxR2, However, the results of GPx activity assay (Fig. 8B) showed SeC and AF alone or in combination didn’t affect the intracellular GPx activities. These results suggest that SeC and AF specifically act on TrxR.

**Figure 9:**
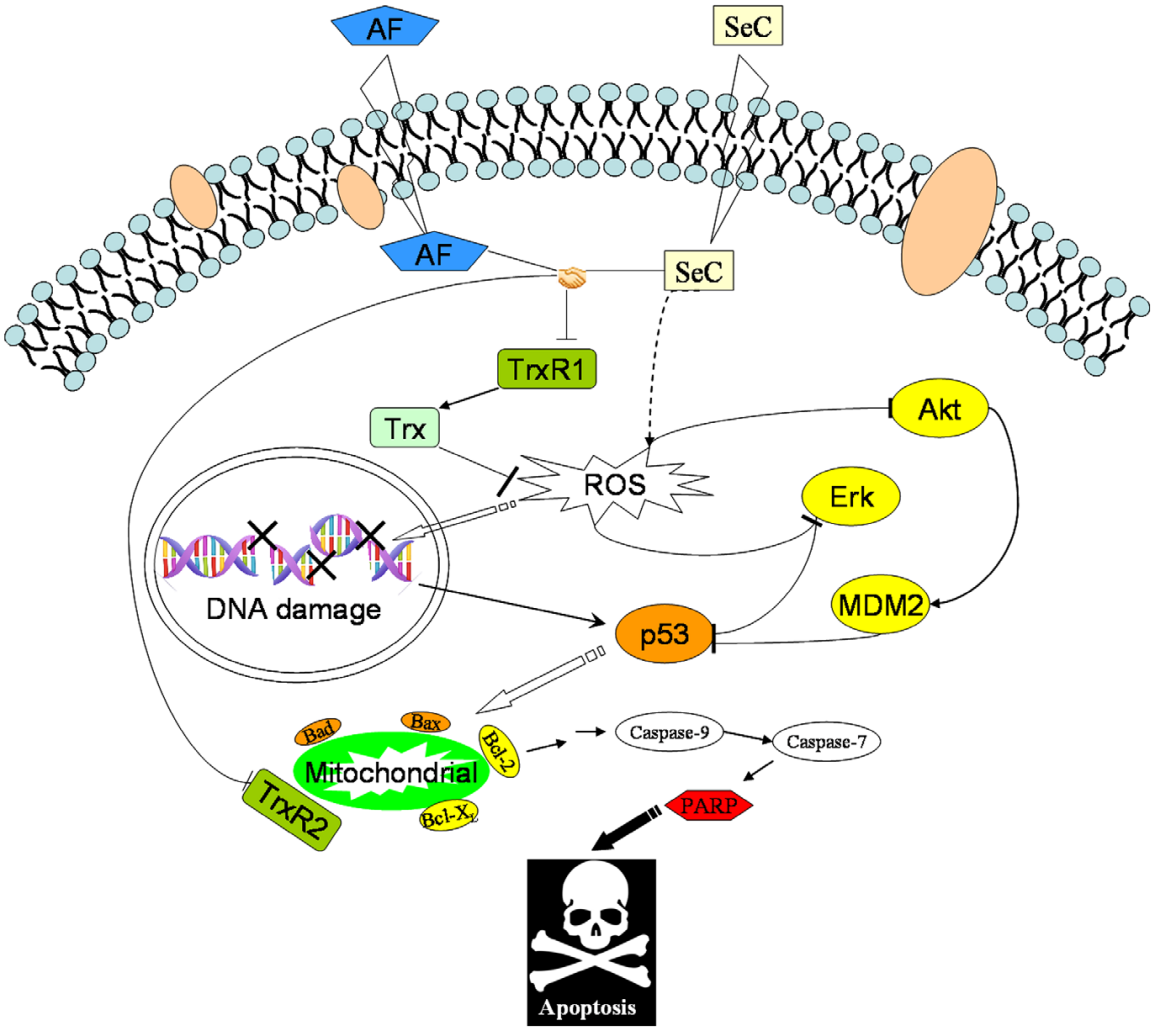
Proposed signaling pathways of apoptosis induced by SeC and AF. AF inhibits the Trx-reduced activity of TrxR, whereas SeC competes with Trx and causes ROS accumulation. AF and SeC synergistically induce DNA damage, AKT and ERK dephosphorylation and result in the activation of p53 pathway, which in turn trigger mitochondrial dysfunction to amplify the apoptotic signals.

Moreover, Western blot analysis was also employed to examine the expression level of TrxR1 and generation of Trx-reduced (Fig. 8C). The results revealed that no significant alteration in the expression level of TrxR1 protein was observed in MCF-7 cells exposed to SeC and/or AF. However, decrease in Trx-reduced was detected in MCF-7 cells exposed to SeC or AF alone, and the down-regulation of Trx-reduced was synergistically enhanced by combination of SeC and AF, which was highly consistent with the tendency of TrxR activity. Interestingly, it was also found that SeC alone inhibited the TrxR activity and Trx-reduced expression in a higher degree by comparing with AF, demonstrating the application potential of SeC to be further developed as an alternative of TrxR inhibitor. Taken together, our results indicate that, SeC and AF synergistically induced cancer cell apoptosis through inhibition of TrxR activity, which led to overproduction of ROS and activation of downstream apoptotic signaling pathways (Fig. 9).

## Discussion

GPx and TrxR are generally expressed in mammal cells, and TrxR is overexpressed in many cancer cells and has been identified as a potential target of anticancer drugs. The expression of TrxR is closely correlated with the resistance of cancer cells to chemotherapeutic agents, such as cisplatin and doxorubicin. Till now, many inhibitors of TrxR have been developed for treatment of tumors, such as gold-containing molecules, aurothioglucose, AF, Se- and tellurium-containing compounds, some natural products and their synthetic analogues [32], [33]. Recent studies have found that AF could bind to the selenocysteine-containing C-terminal and the N-terminal redox center of TrxR to inhibit its activity [33]. Moreover, Se compounds could probably act as substrates to compete with Trx, and resulted in the consumption of Trx-reduced [31], [32], [34], [35], [36], [37]. In the present study, we showed that, SeC and AF could cooperatively inhibit the TrxR1 and TrxR2 activity and Trx-reduced generation specially. To our knowledge, it is the first study to demonstrate that SeC could cooperate with AF to induce apoptosis in cancer cells by causing an enhanced effect on TrxR inhibition. Our finding provides important information about the improvement of the therapeutic efficacy of current anticancer drugs.

Trx-mediated redox regulation is involved in many physiological or pathological signaling pathways [38]. Intracellular Trx system could regulate the DNA and protein synthesis, repair, and redox balance by counteracting excess ROS [39]. We and others have showed that the apoptosis-inducing activities of Se were caused by induction of oxidative stress and disruption of redox homeostasis [14], [40]. ROS, including the superoxide anion, hydrogen peroxide and hydroxyl radical, are produced under normal aerobic growth conditions within the cells, but they could be elevated under the influence of external stimuli. Intracellular ROS may attack the lipids in cell membrane, proteins and DNA, and cause oxidative injury. In the present study, we showed that, SeC and AF cooperatively induced the accumulation of intracellular ROS generation, which was consistent with the change of Trx and TrxR. Furthermore, pretreatment of the cells with ROS scavengers (NAC and GSH) almost completely block the downstream apoptotic signals, suggesting that ROS is a crucial factor that regulates the cell apoptosis induced by SeC and AF.

Several protein kinases have been known to regulate cell proliferation and survival. MAPKs and PI3K/AKT pathways are the major oxidative stress sensitive signal transduction pathways in most cell types [29], [41]. MAPKs, including c-Jun NH2-terminal protein kinase/stress activated protein kinases (JNK/SAPKs), p38 MAPK, and extracellular signal-regulated kinase (ERK), have been found implicated in apoptosis and cell cycle regulation in diverse cell models [29], [30], [41]. In general, JNK and p38 MAPK are activated by diverse stimuli, such as oxidative stress, UV irradiation and osmotic shock [30]. In contrast, ERK plays vital roles in cell growth and division and is generally considered to be a prosurvival mediator [42]. Beside MAPKs, AKT is also able to mediate cell growth via the phosphorylation of a variety of downstream substrates, including Bad, glycogen synthase kinase 3β (GSK3β), and FOXO transcription factor [43]. Many studies have demonstrated the involvement of MAPK and PI3K/AKT pathways in apoptosis and/or cell cycle arrest in human cancer cells induced by selenocompounds [43], [44]. Studies also showed that inhibition of AKT phosphorylation by methylseleninic acid or Se-methylselenocysteine induced cancer cell apoptosis or enhanced the apoptosis-inducing effects of chemotherapeutic drugs [45], [46]. However, limited information is available on the significance of MAPK and PI3K/AKT pathway as potential targets for the chemosensitization effects of SeC. The present study displayed the synergistic anticancer action of SeC and AF with to the involvement of PI3K/AKT and MAPK pathways. We showed that SeC caused the cell apoptosis through dephosphorylation of ERK and AKT. Inhibitors of ERK and AKT significantly enhanced the SeC and AF-induced apoptosis in MCF-7 cells, indicating that ERK and AKT were critical in mediating SeC and AF-induced growth inhibition. Collectively, our results suggest the important roles of ERK and AKT in regulating apoptosis induced by SeC and AF.

P53 is a transcription factor that could directly or indirectly induce cell apoptosis through both the extrinsic and intrinsic apoptotic pathways [47]. P53 could be regulated via various signaling pathways, including phosphorylation of MAPKs, AKT, MDM2 and ROS-caused DNA damage [48]. The function and activity of p53 is regulated through transcription, translation, protein–protein interactions with cooperating factors, and extensive post-translational modifications, such as phosphorylation and acetylation on specific amino acids [14]. The pro-apoptotic function of p53 was also considered to be associated with mitochondrial release of apoptogenic factors, such as cytochrome c and SMAC [49]. We have previously showed that p53 activation was an upstream cellular event that led to mitochondrial dysfunction [50]. Growing evidence suggested that p53-mediated apoptosis was activated by DNA strand breaks (DSBs), DNA intra-strand adducts, and DNA–protein crosslinks [50]. In the current study, we found that, the combination of SeC and AF significantly caused the p53 phosphorylation without any change in total p53 protein. Moreover, phospho-Histone H2A.X, a marker of DNA damage, was also detected with similar trend, suggesting that the involvement of DNA damage and p53 phosphorylation in cell apoptosis. Furthermore, down-regulation of phospho-MDM2 that could specifically bind to p53 and lead to degradation of p53 via ubiquitination pathway, was also observed in the treated cells. Taken together, the overproduction of ROS induced by SeC and AF could lead to AKT and ERK dephosphorylation, and DNA damage, which subsequently triggered p53 activation. The apoptotic signal was amplified and transduced to the mitochondria and downstream effectors.

As far as caspases are concerned, two well characterized caspase-mediated pathways have been reported to regulate apoptosis in cancer cells exposed to many chemotherapeutic drugs [51]. Generally, the mitochondrial (intrinsic) pathway involves mitochondrial permeability transition and the release of cytochrome c into cytosol. The cytosolic cytochrome c then forms a protein complex known as the apoptosome, leading to the activation of caspase-9, which in turn cleaves and activates the effector caspases, such as caspases-3 and -7, and PARP cleavage. The death receptor (extrinsic) pathway involves the engagement of the death receptors, which recruit the adapter protein FADD and procaspase-8, thereby forming a complex known as the death-inducing signaling complex. The consequent proximity of caspase-8 proteins in the death-inducing signaling complex allows their auto-cleavage and activation. Studies have found that the AF-induced apoptosis in cancer cells was mainly caused by activation of the mitochondria-mediated pathway with alteration of Bcl-2 family protein and release of cytochrome c [52], [53]. In another hand, our previous studies have demonstrated that SeC induced apoptosis in MCF-7 cells with the involvement of depletion of ΔΨm, regulation of Bcl-2 family proteins and release of apoptotic factors [14]. In the current study, we showed that, SeC in combination with AF resulted in significantly enhanced activation of caspases-7 and caspase-9, PARP cleavage, mitochondrial dysfunction, up-regulation of Bax, Bad and down-regulation of Bcl-2. These results suggest the important role of the mitochondria-mediated apoptotic pathway in the synergistic effects of SeC and AF in cancer cells.

In summary, the combination of SeC and AF synergistically inhibited the growth of human breast cancer cells through induction of apoptosis by targeting TrxR (Fig. 9). ROS acted as an upstream mediator for AKT and ERK dephosphorylation, and triggered cell DNA damage, which led to activation of p53 pathway. On the other hand, p53 activation enhanced the ROS generation through induction of mitochondrial dysfunction. Taken together, our results suggest that the combination of SeC and AF could be a novel strategy to achieve anticancer synergy by targeting TrxR system.
