# Expression of concern: Enhancement of auranofin-induced apoptosis in MCF-7 human breast cells by selenocystine, a synergistic inhibitor of thioredoxin reductase

**DOI:** 10.1371/journal.pone.0337853

**Published:** 2025-12-01

**Authors:** The PLOS One Editors

Following the publication of this article concerns were raised regarding results presented in Figs 6, 7, and 8. Specifically,

- The Fig 6C phospho-ERK and total ERK panels appear to partially overlap when rotated.
- The Fig 7C phospho-ERK and total ERK panels appear to partially overlap when flipped.
- The Fig 8C β-actin panel of this article [1], and the Fig 2D, 3A, and 5B β-actin panels of [2,3] appear similar despite being used to represent different experimental conditions.

The authors did not agree with the journal’s observations for Figs 6C and 7C, and stated that an incorrect panel was used for the preparation of Fig 8C. The authors provided the original blot data underlying the published panels in Figs 6A, 6C, 7C and 8C (S1-S4 Files), the individual-level data underlying the published graphs (S5–10 Files), and an updated Fig 8C presenting the correct β-actin panel. The data provided did not fully resolve the journal’s concerns with Figs 6C and 7C and raised additional methodological concerns calling into question the reliability of the phosphorylated blot results.

The *PLOS One* Editors issue this Expression of Concern to inform readers that the western blot results presented in Figs 6 and 7 should be interpreted with caution and to share the underlying data provided by the authors.

**Fig 8:**
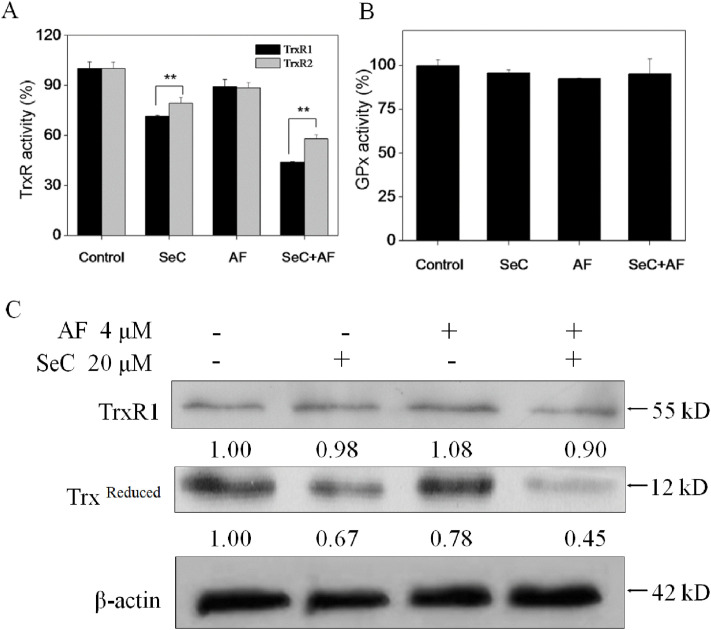
Changes of antioxidant enzyme activities of TrxR and GPx in MCF-7 cells induced by SeC and AF. Enzymatic activity of TrxR1, TrxR2 (**A**) and GPx (**B**) in MCF-7 cells after treatment with SeC or/and AF. Cells were pretreated with or without 20 µM SeC for 24 h and then cultured in the presence or absence of 4 µM AF for 6 h. Bars with different characters are statistically different at *P*<0.01. (**C**) Cell lysates were subjected to Western blot analysis, protein levels of TrxR1 and Redox thioredoxin were examined. Equal protein loading was confirmed by analysis of β-actin in the protein extracts. Similar results were obtained from three independent experiments.

## Supporting information

S1 File Underlying blot data for Fig 6A. (PPTX)

S2 File Underlying blot data for Fig 6C. (PPTX)

S3 File Underlying blot data for Fig 7C. (PPTX)

S4 File Underlying blot data for Fig 8C. (PPTX)

S5 File Individual level data underlying Fig 1A. (XLSX)

S6 File Individual level data underlying Fig 5B. (XLSX)

S7 File Individual level data underlying Fig 6B. (XLSX)

S8 File Individual level data underlying Fig 7A. (XLSX)

S9 File Individual level data underlying Fig 8A. (XLSX)

S10 File Individual level data underlying Fig 8B. (XLSX)
