# Supplementary material for: Expression of concern: Enhancement of auranofin-induced apoptosis in MCF-7 human breast cells by selenocystine, a synergistic inhibitor of thioredoxin reductase
Source: PLoS One. 2025 Dec 1;20(12):e0337853. doi: 10.1371/journal.pone.0337853 (PMC12668561; doi:10.1371/journal.pone.0337853)
Supplement: S1 File — (PPTX) [file pone.0337853.s001.pptx]

## Slide 1
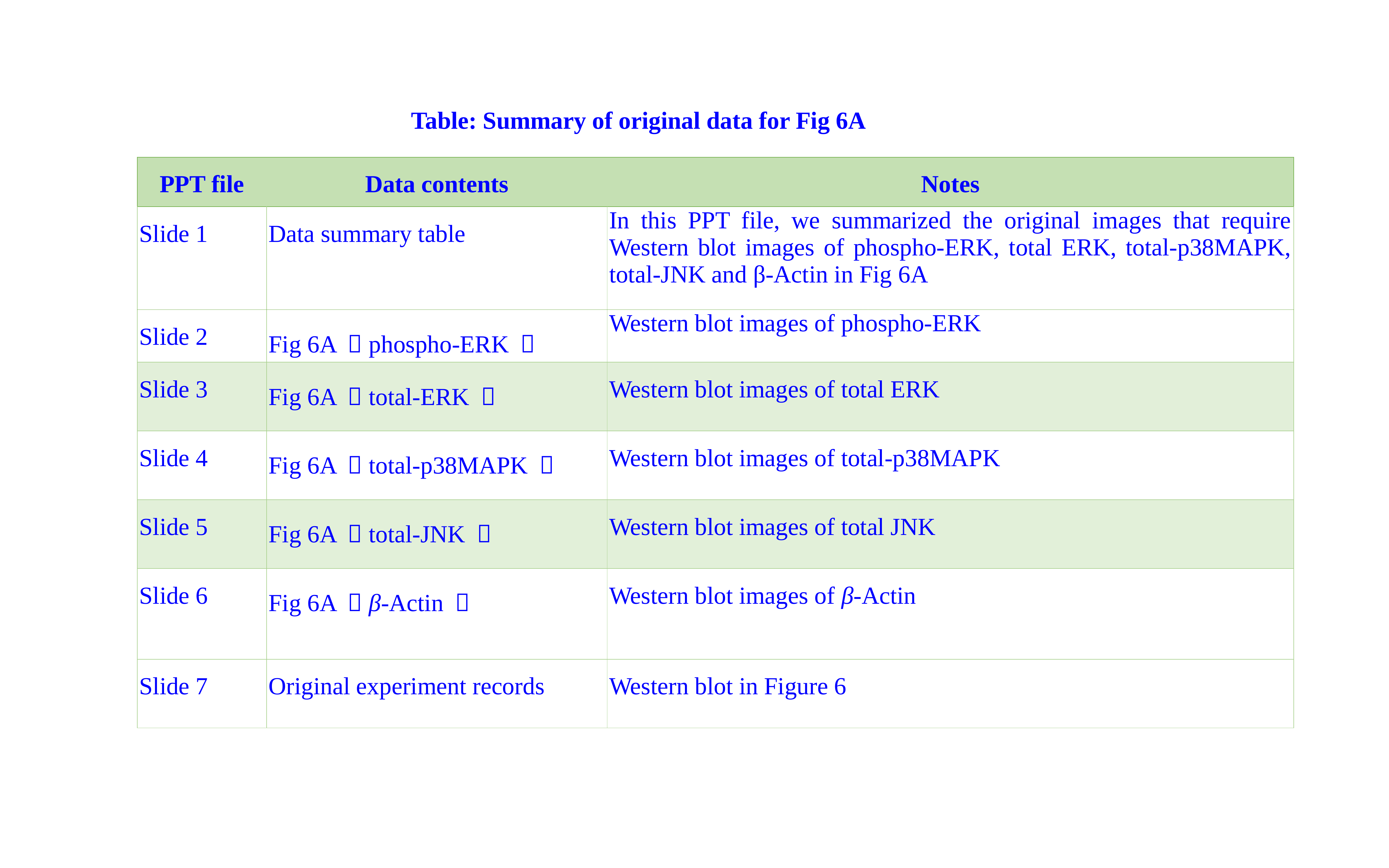

Table: Summary of original data for Fig 6A
| PPT file | Data contents | Notes |
| --- | --- | --- |
| Slide 1 | Data summary table | In this PPT file, we summarized the original images that require Western blot images of phospho-ERK, total ERK, total-p38MAPK, total-JNK and β-Actin in Fig 6A |
| Slide 2 | Fig 6A （phospho-ERK ） | Western blot images of phospho-ERK |
| Slide 3 | Fig 6A （total-ERK ） | Western blot images of total ERK |
| Slide 4 | Fig 6A （total-p38MAPK ） | Western blot images of total-p38MAPK |
| Slide 5 | Fig 6A （total-JNK ） | Western blot images of total JNK |
| Slide 6 | Fig 6A （β-Actin ） | Western blot images of β-Actin |
| Slide 7 | Original experiment records | Western blot in Figure 6 |

## Slide 2
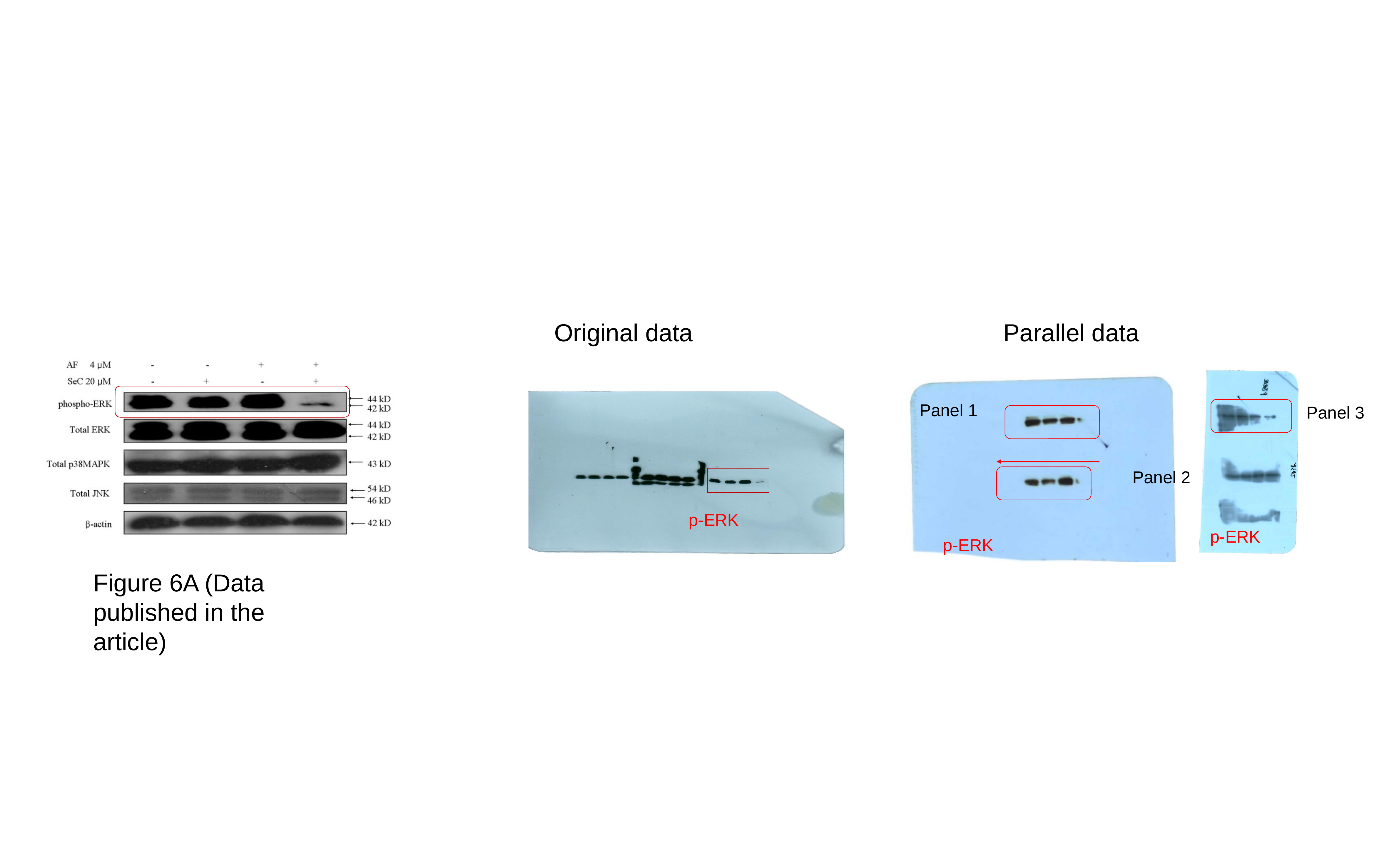

Original data
Parallel data
Panel 1
Panel 3
Panel 2
p-ERK
p-ERK
p-ERK
Figure 6A (Data published in the article)

## Slide 3
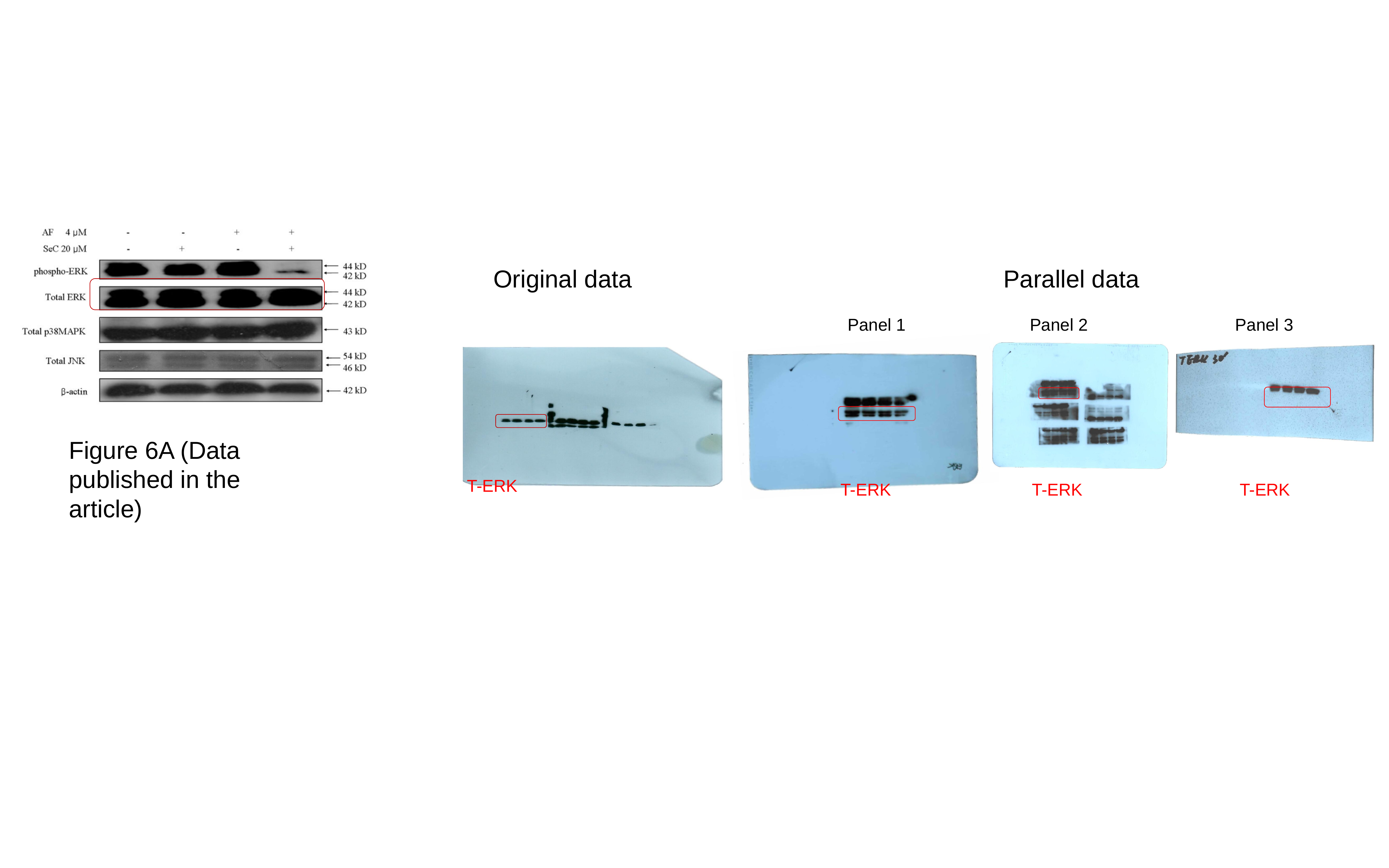

Original data
Parallel data
Panel 1
Panel 2
Panel 3
Figure 6A (Data published in the article)
T-ERK
T-ERK
T-ERK
T-ERK

## Slide 4
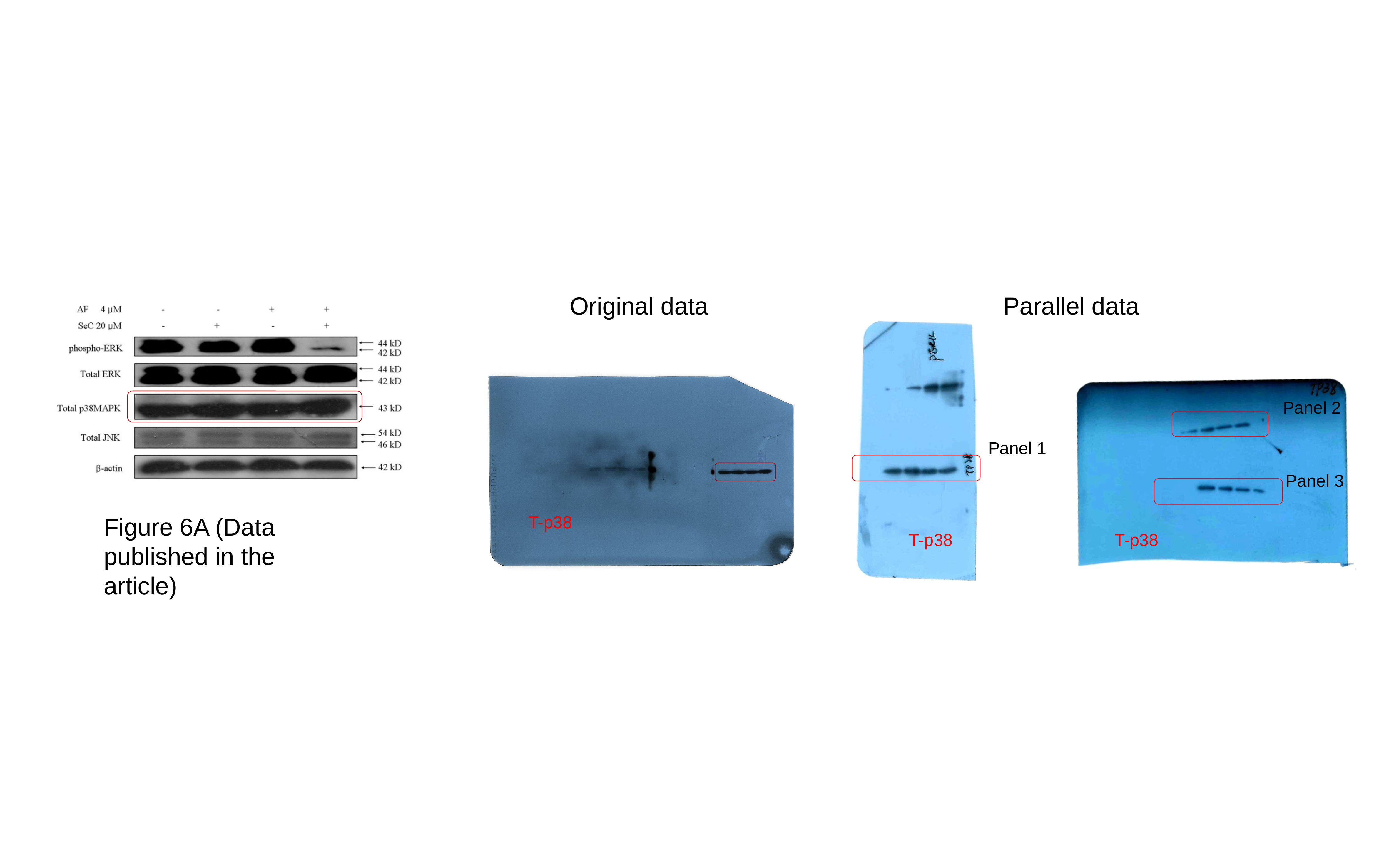

Original data
Parallel data
Panel 2
Panel 1
Panel 3
T-p38
T-p38
Figure 6A (Data published in the article)
T-p38

## Slide 5
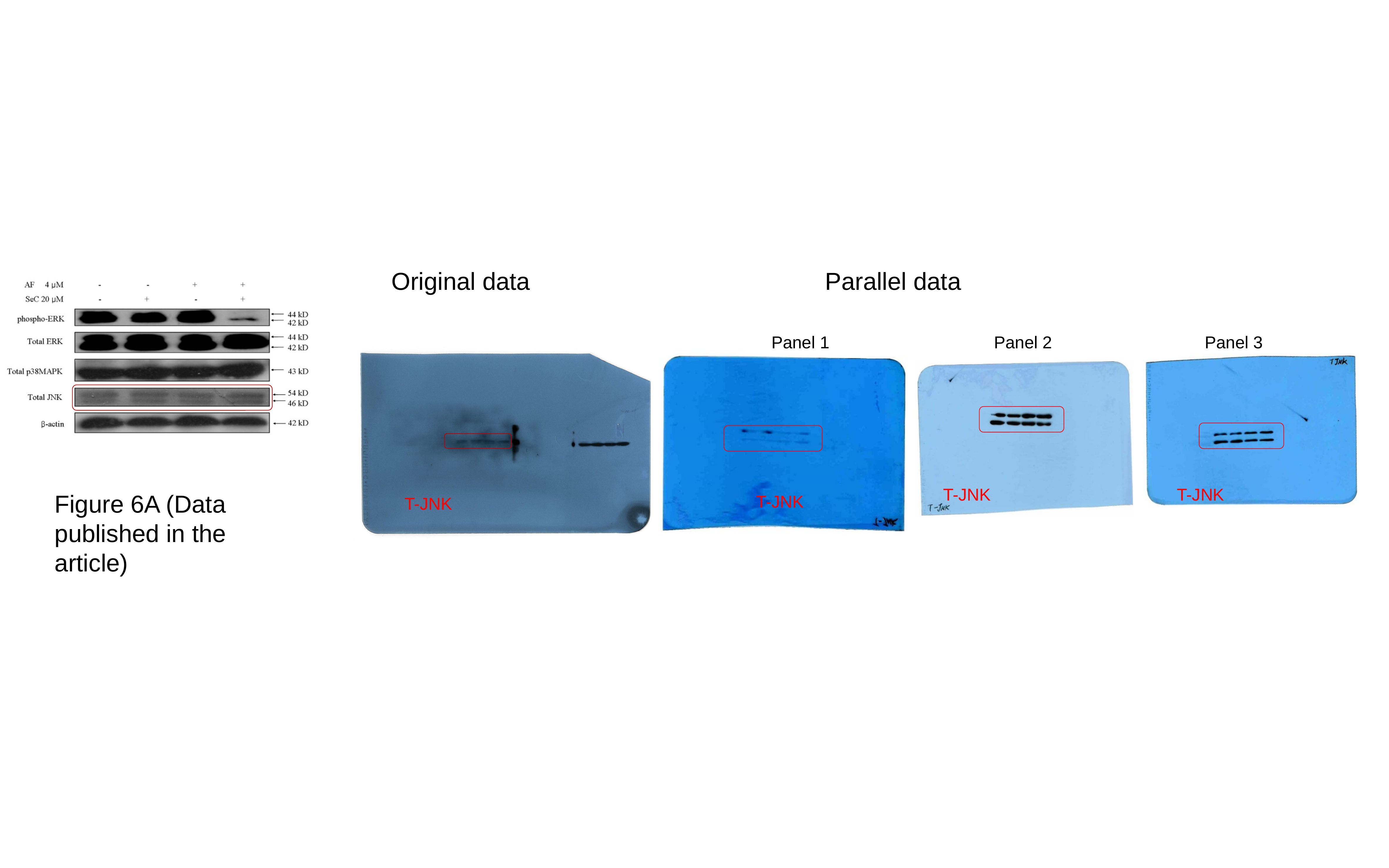

Original data
Parallel data
Panel 1
Panel 2
Panel 3
T-JNK
T-JNK
T-JNK
Figure 6A (Data published in the article)
T-JNK

## Slide 6
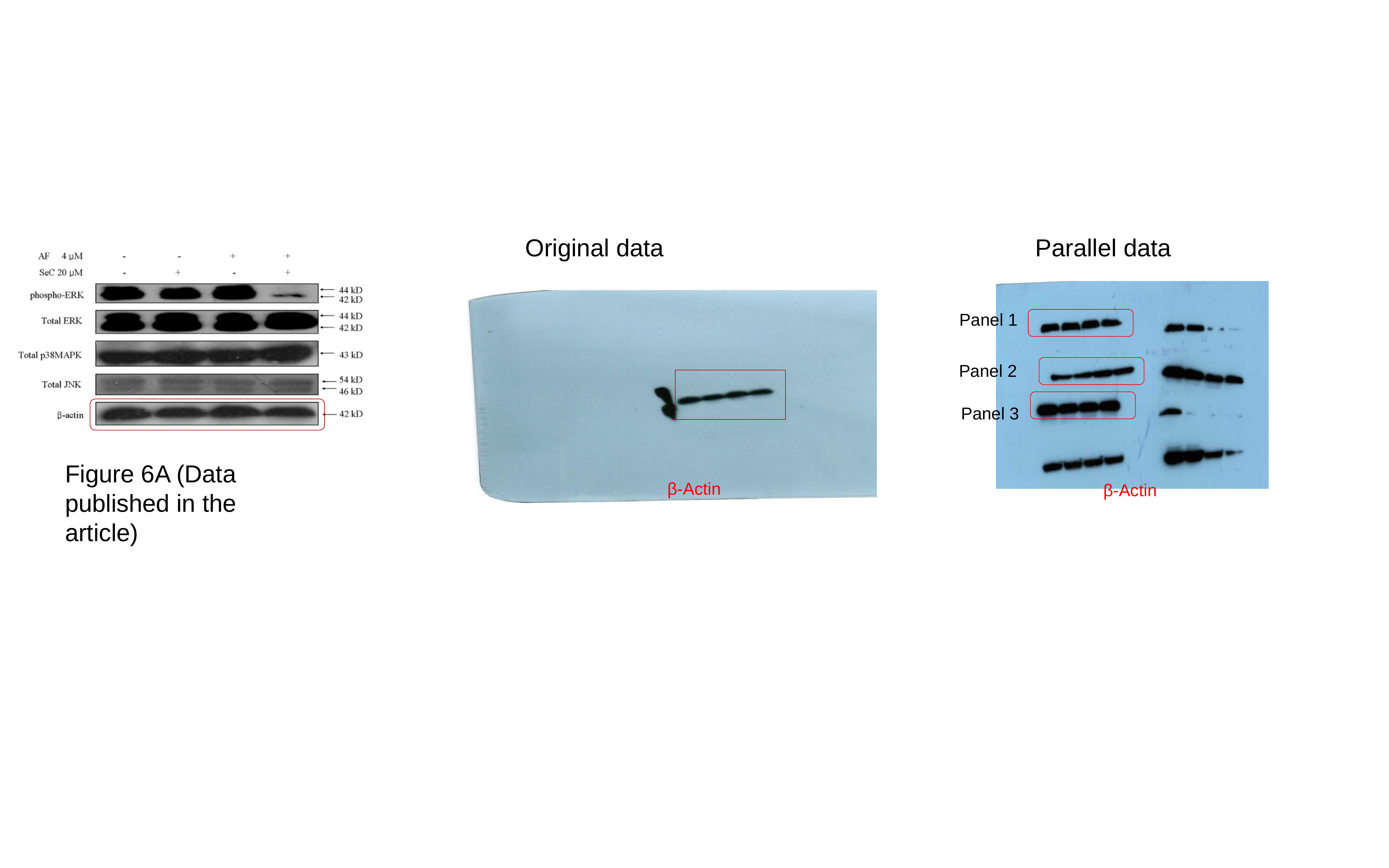

Original data
Parallel data
Panel 1
Panel 2
Panel 3
Figure 6A (Data published in the article)
β-Actin
β-Actin

## Slide 7
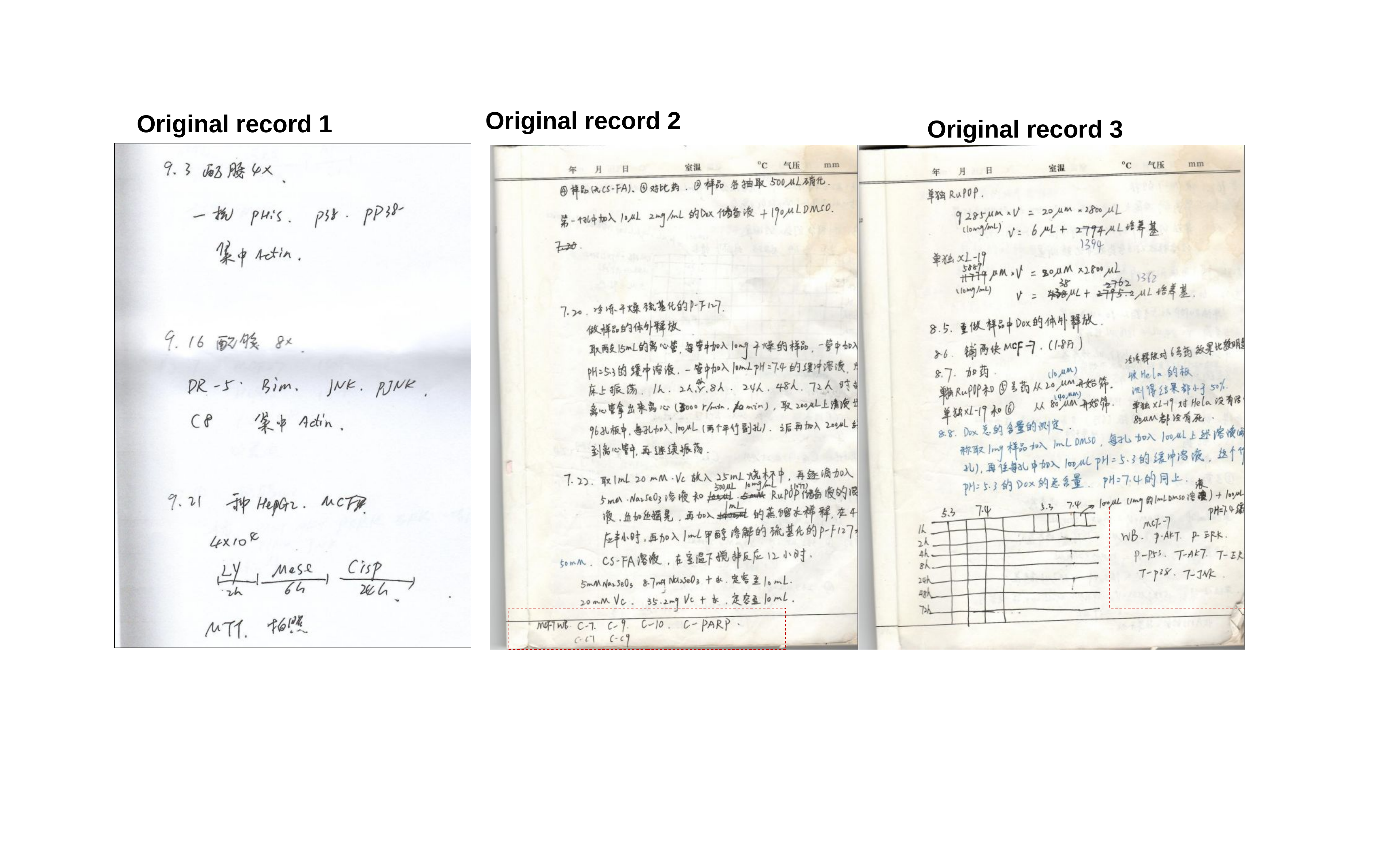

Original record 2
Original record 1
Original record 3
