# Supplementary material for: Expression of concern: Enhancement of auranofin-induced apoptosis in MCF-7 human breast cells by selenocystine, a synergistic inhibitor of thioredoxin reductase
Source: PLoS One. 2025 Dec 1;20(12):e0337853. doi: 10.1371/journal.pone.0337853 (PMC12668561; doi:10.1371/journal.pone.0337853)
Supplement: S3 File — (PPTX) [file pone.0337853.s003.pptx]

## Slide 1
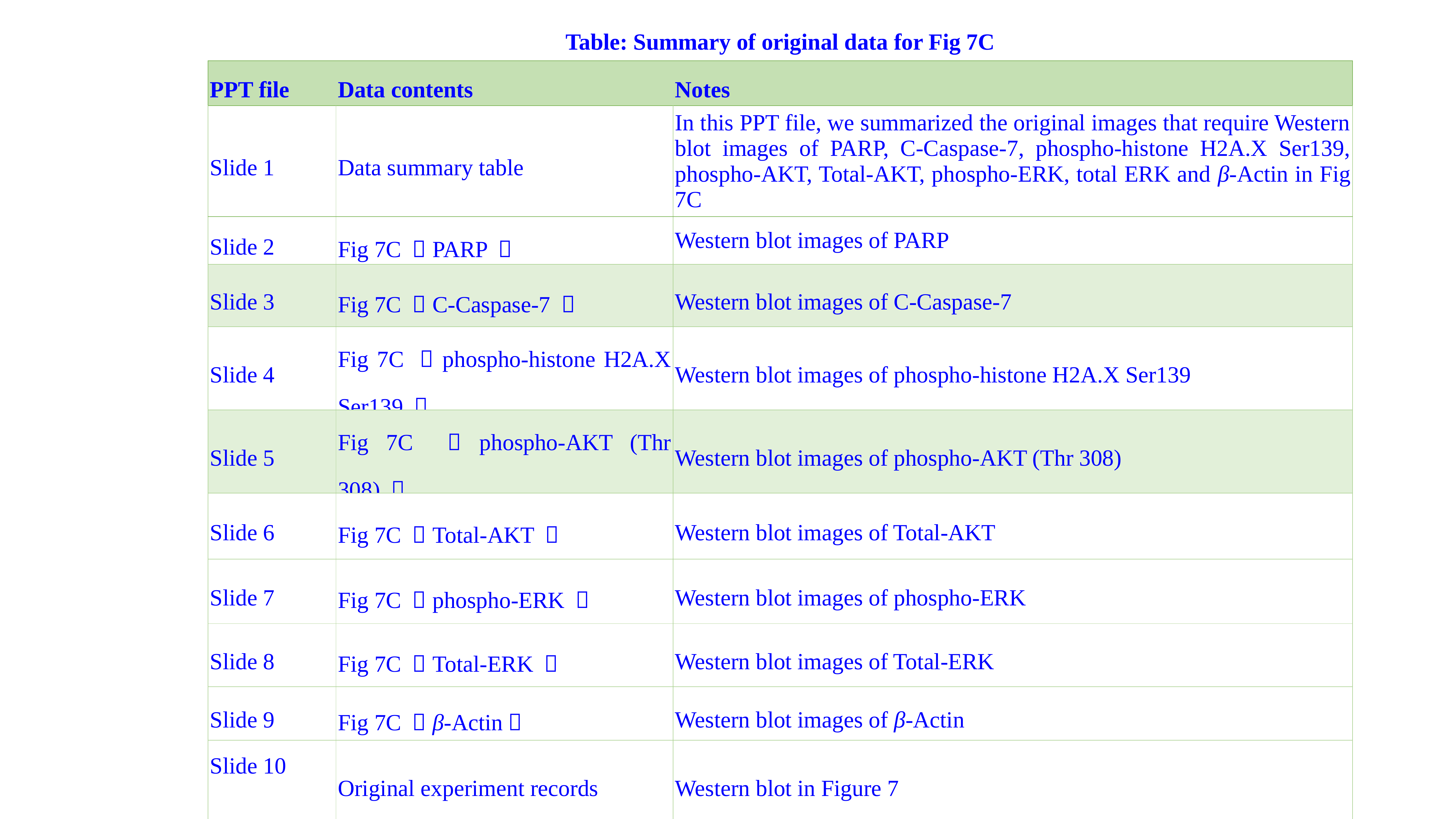

Table: Summary of original data for Fig 7C
| PPT file | Data contents | Notes |
| --- | --- | --- |
| Slide 1 | Data summary table | In this PPT file, we summarized the original images that require Western blot images of PARP, C-Caspase-7, phospho-histone H2A.X Ser139, phospho-AKT, Total-AKT, phospho-ERK, total ERK and β-Actin in Fig 7C |
| Slide 2 | Fig 7C （PARP ） | Western blot images of PARP |
| Slide 3 | Fig 7C （C-Caspase-7 ） | Western blot images of C-Caspase-7 |
| Slide 4 | Fig 7C （phospho-histone H2A.X Ser139 ） | Western blot images of phospho-histone H2A.X Ser139 |
| Slide 5 | Fig 7C （phospho-AKT (Thr 308) ） | Western blot images of phospho-AKT (Thr 308) |
| Slide 6 | Fig 7C （Total-AKT ） | Western blot images of Total-AKT |
| Slide 7 | Fig 7C （phospho-ERK ） | Western blot images of phospho-ERK |
| Slide 8 | Fig 7C （Total-ERK ） | Western blot images of Total-ERK |
| Slide 9 | Fig 7C （β-Actin） | Western blot images of β-Actin |
| Slide 10 | Original experiment records | Western blot in Figure 7 |

## Slide 2
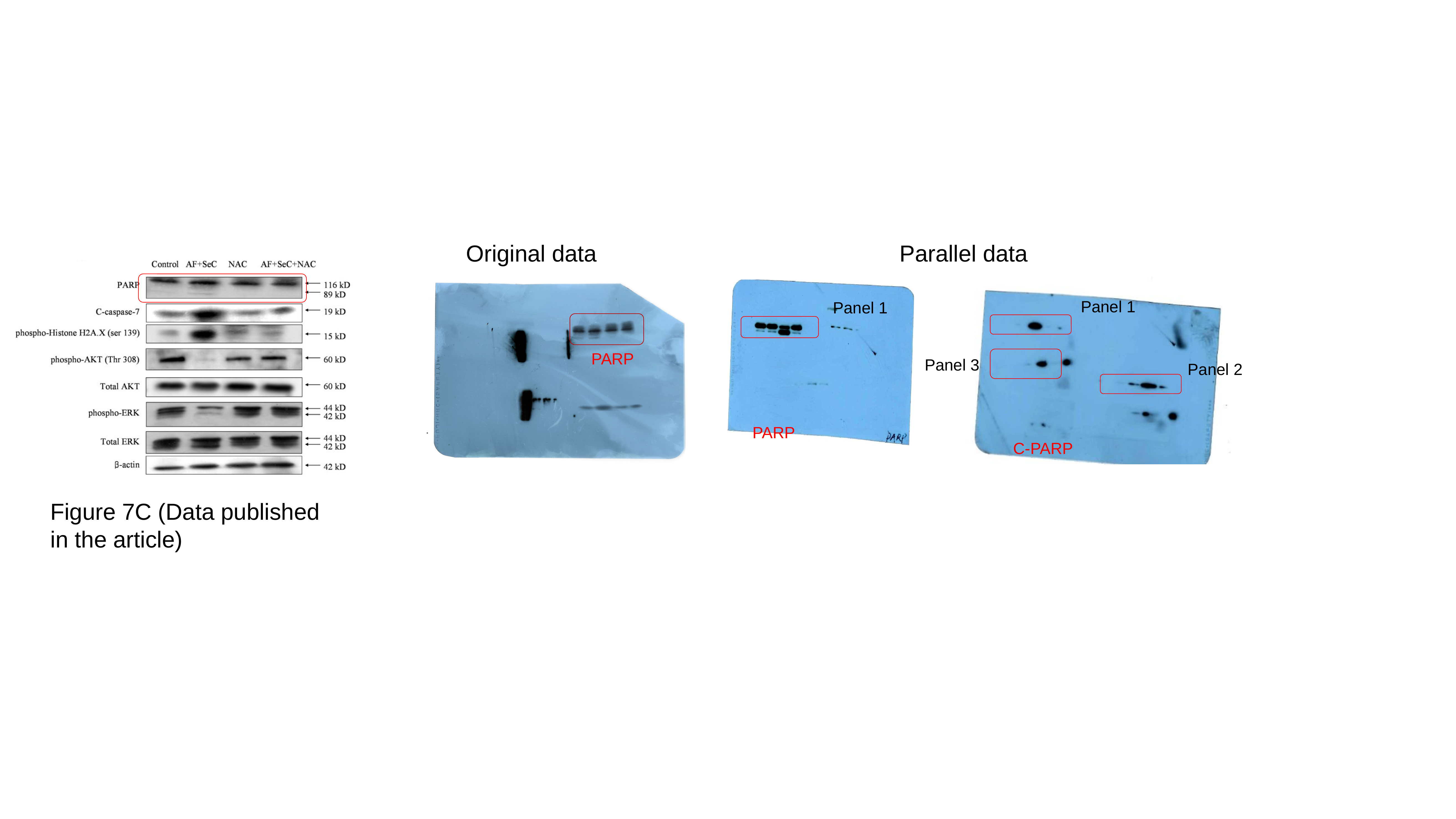

Original data
Parallel data
Panel 1
Panel 1
PARP
Panel 3
Panel 2
PARP
C-PARP
Figure 7C (Data published in the article)

## Slide 3
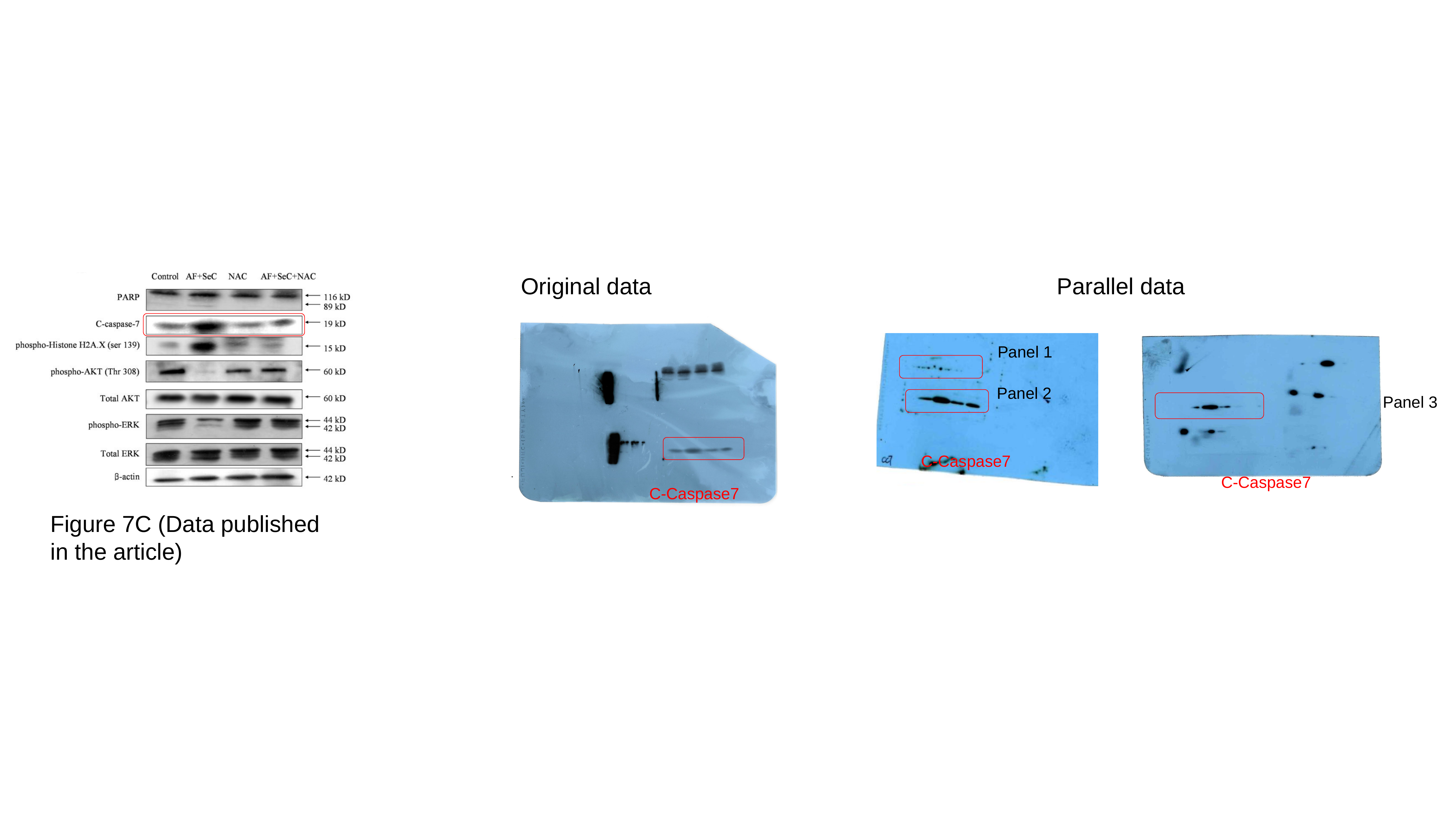

Original data
Parallel data
Panel 1
Panel 2
Panel 3
C-Caspase7
C-Caspase7
C-Caspase7
Figure 7C (Data published in the article)

## Slide 4
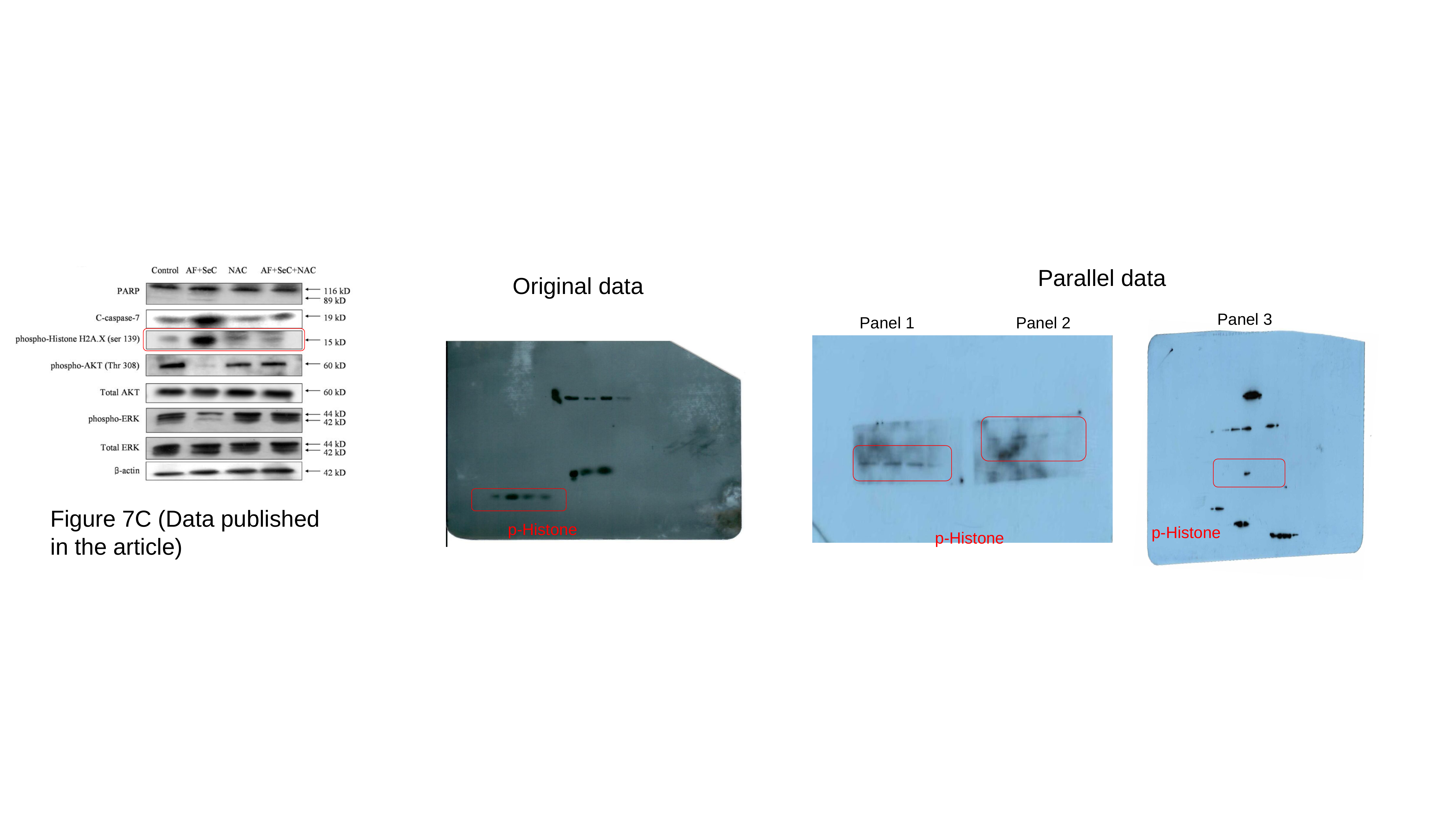

Parallel data
Original data
Panel 3
Panel 1
Panel 2
Figure 7C (Data published in the article)
p-Histone
p-Histone
p-Histone

## Slide 5
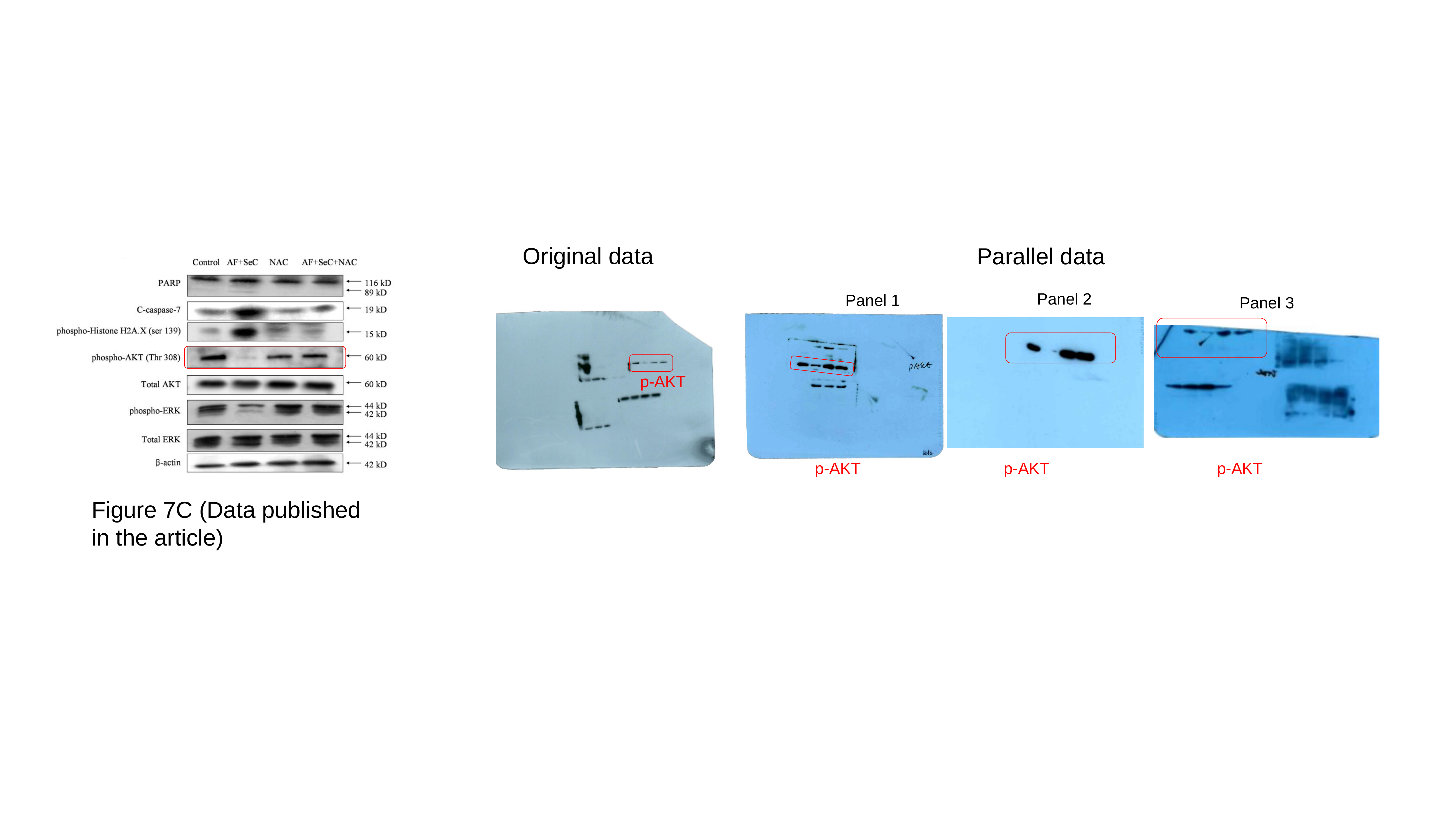

Original data
Parallel data
Panel 2
Panel 1
Panel 3
p-AKT
p-AKT
p-AKT
p-AKT
Figure 7C (Data published in the article)

## Slide 6
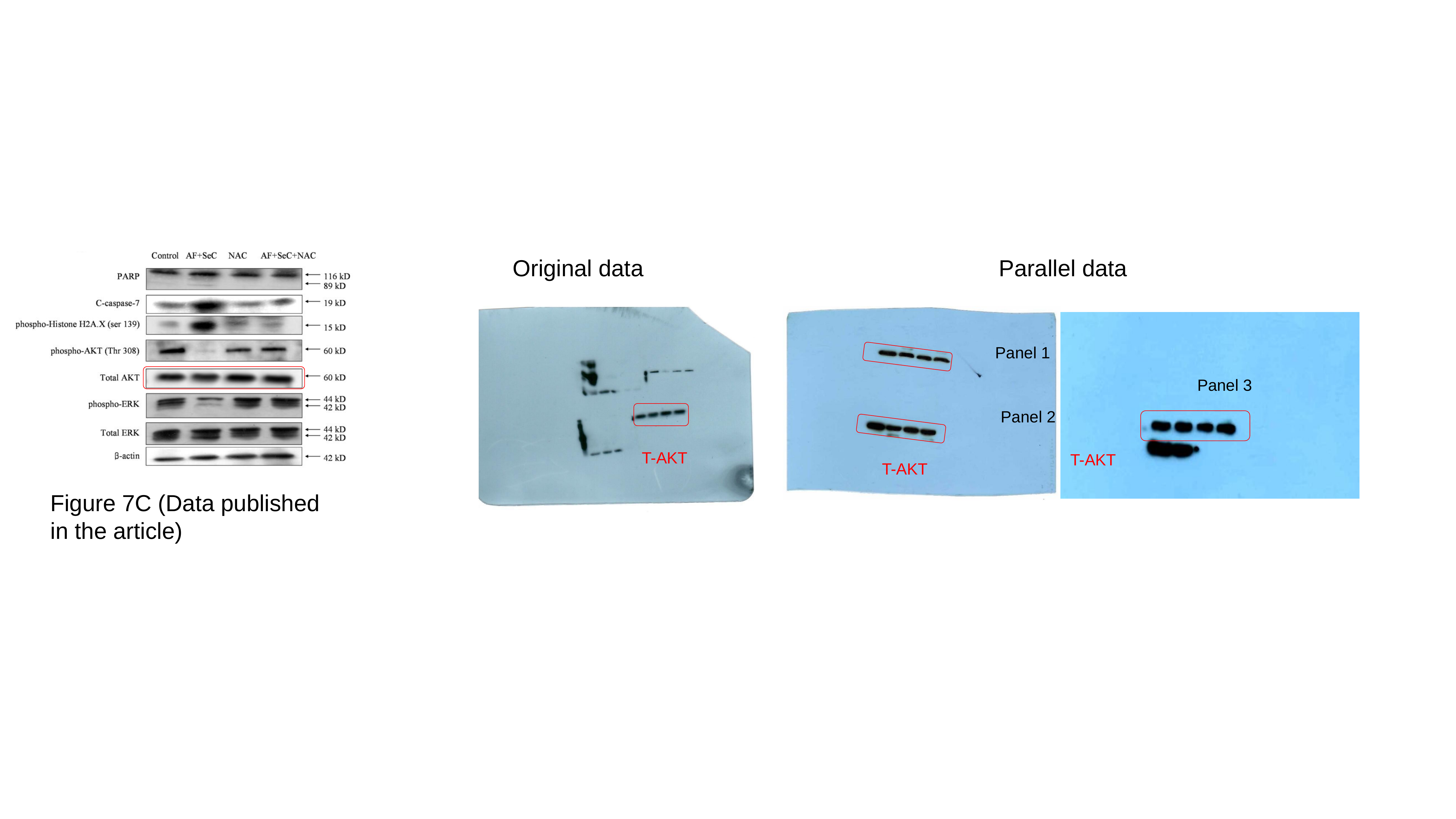

Original data
Parallel data
Panel 1
Panel 3
Panel 2
T-AKT
T-AKT
T-AKT
Figure 7C (Data published in the article)

## Slide 7
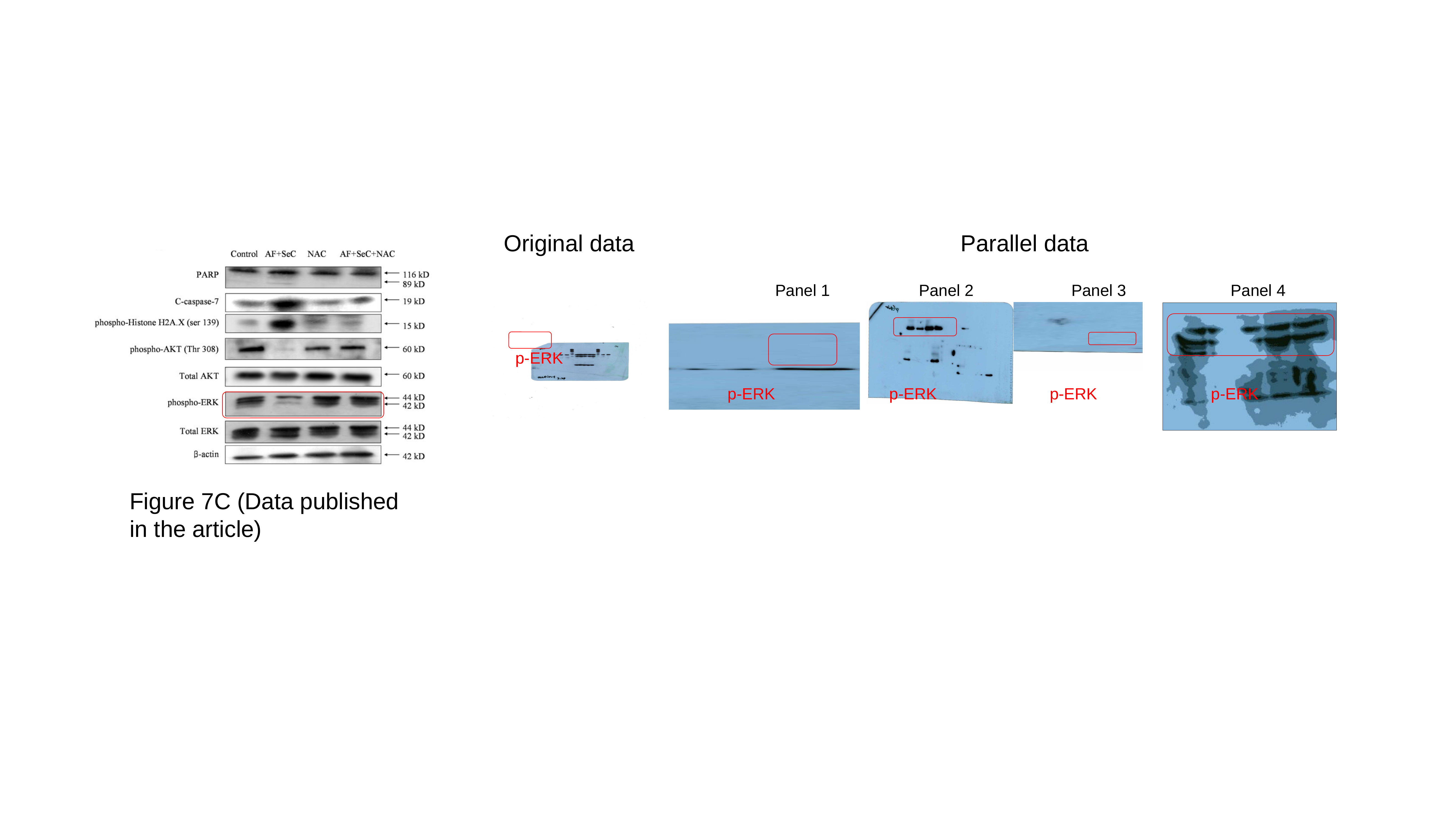

Original data
Parallel data
Panel 1
Panel 2
Panel 3
Panel 4
p-ERK
p-ERK
p-ERK
p-ERK
p-ERK
Figure 7C (Data published in the article)

## Slide 8
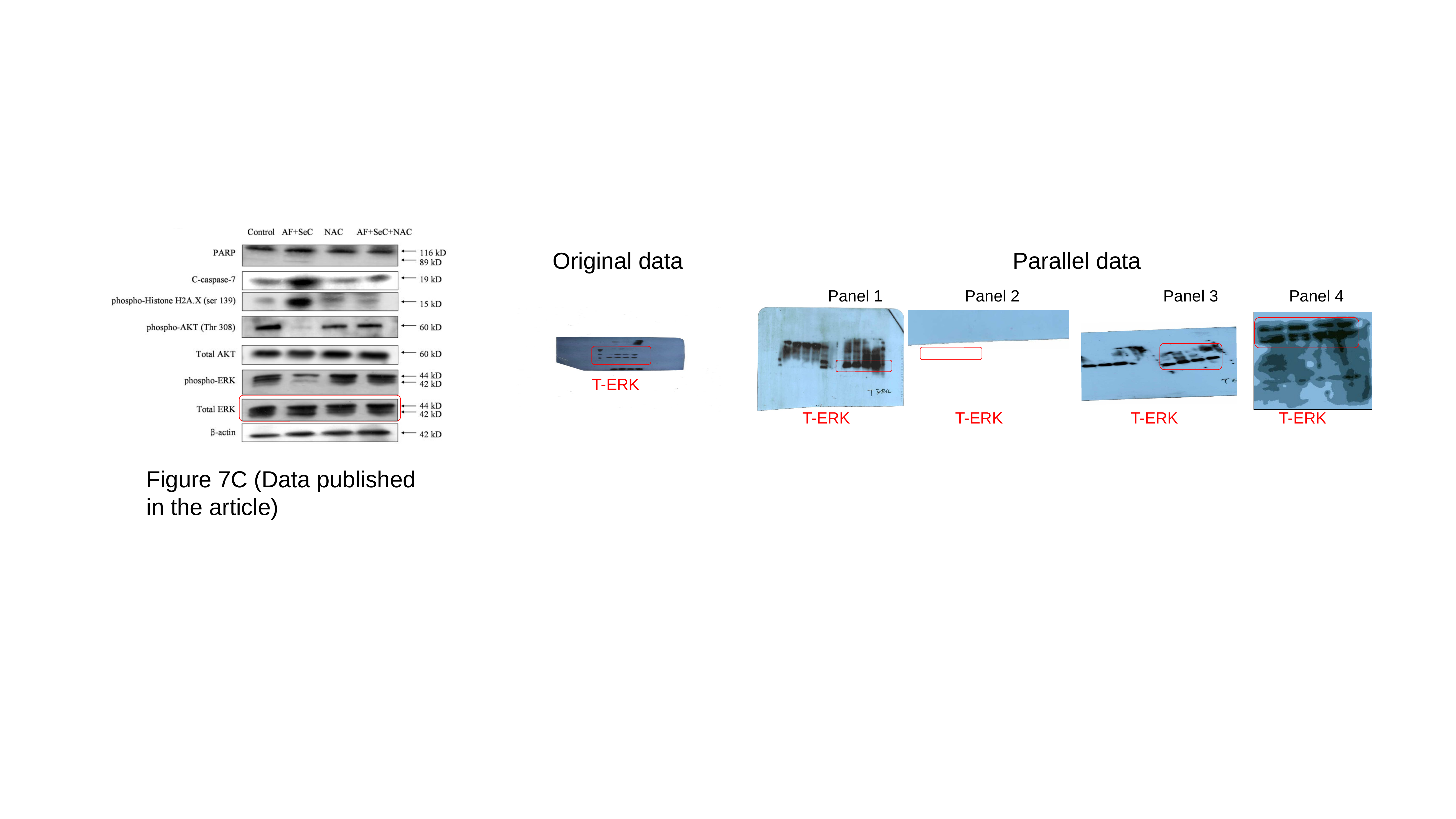

Original data
Parallel data
Panel 1
Panel 2
Panel 3
Panel 4
T-ERK
T-ERK
T-ERK
T-ERK
T-ERK
Figure 7C (Data published in the article)

## Slide 9
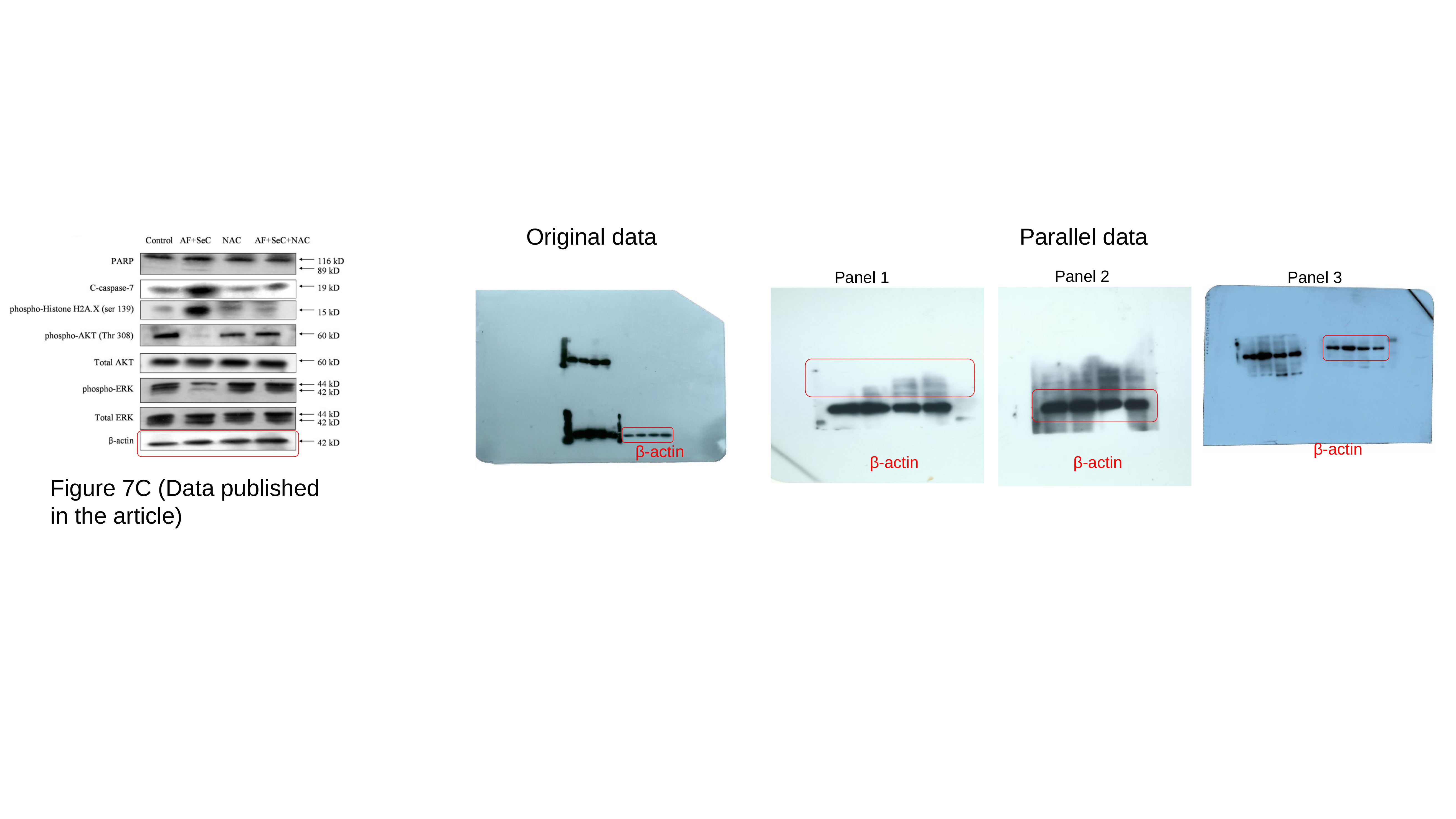

Original data
Parallel data
Panel 2
Panel 1
Panel 3
β-actin
β-actin
β-actin
β-actin
Figure 7C (Data published in the article)

## Slide 10
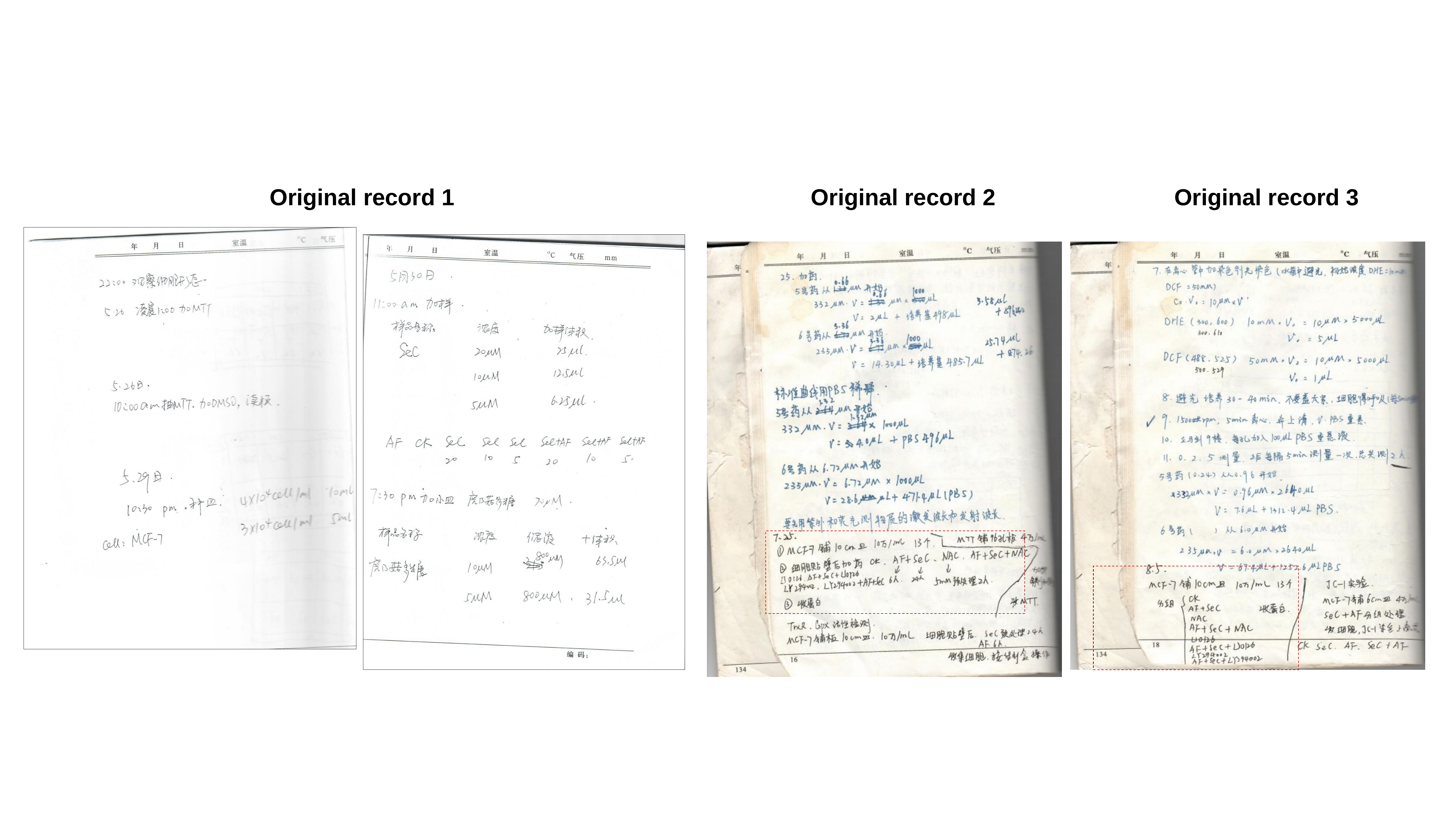

Original record 1
Original record 2
Original record 3
