# Supplementary material for: Expression of concern: Enhancement of auranofin-induced apoptosis in MCF-7 human breast cells by selenocystine, a synergistic inhibitor of thioredoxin reductase
Source: PLoS One. 2025 Dec 1;20(12):e0337853. doi: 10.1371/journal.pone.0337853 (PMC12668561; doi:10.1371/journal.pone.0337853)
Supplement: S4 File — (PPTX) [file pone.0337853.s004.pptx]

## Slide 1
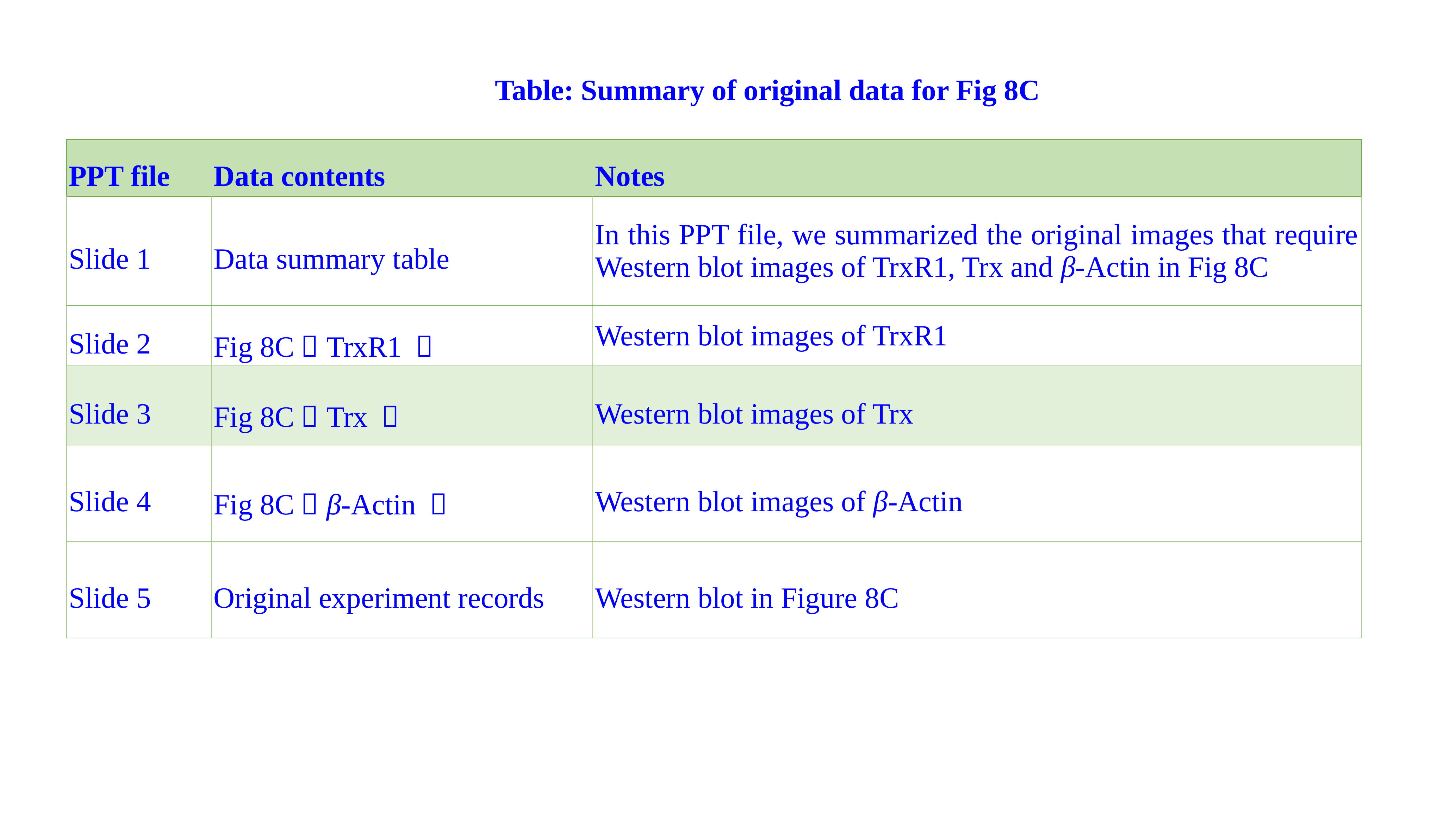

Table: Summary of original data for Fig 8C
| PPT file | Data contents | Notes |
| --- | --- | --- |
| Slide 1 | Data summary table | In this PPT file, we summarized the original images that require Western blot images of TrxR1, Trx and β-Actin in Fig 8C |
| Slide 2 | Fig 8C（TrxR1 ） | Western blot images of TrxR1 |
| Slide 3 | Fig 8C（Trx ） | Western blot images of Trx |
| Slide 4 | Fig 8C（β-Actin ） | Western blot images of β-Actin |
| Slide 5 | Original experiment records | Western blot in Figure 8C |

## Slide 2
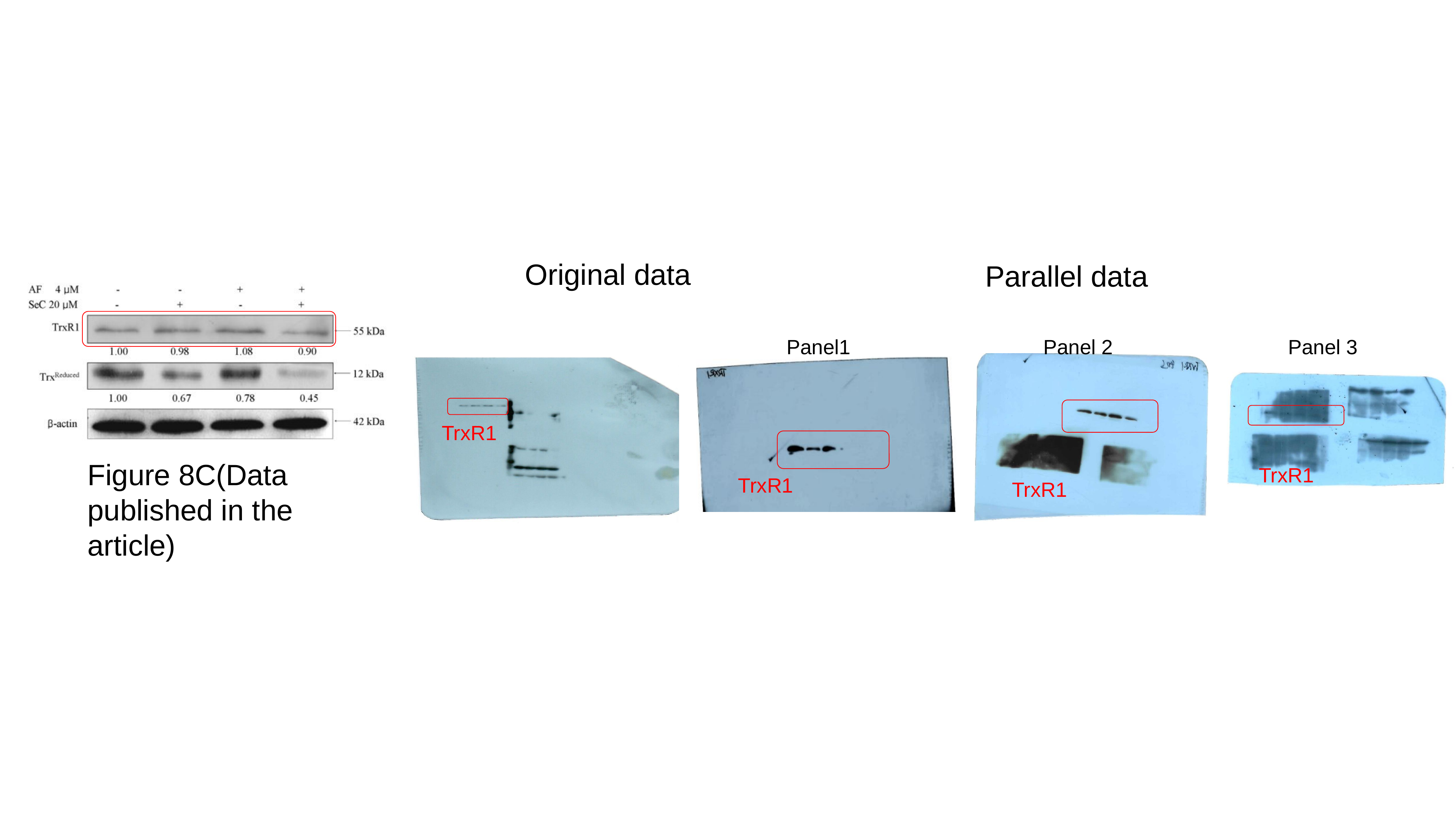

Original data
Parallel data
Panel1
Panel 2
Panel 3
TrxR1
Figure 8C(Data published in the article)
TrxR1
TrxR1
TrxR1

## Slide 3
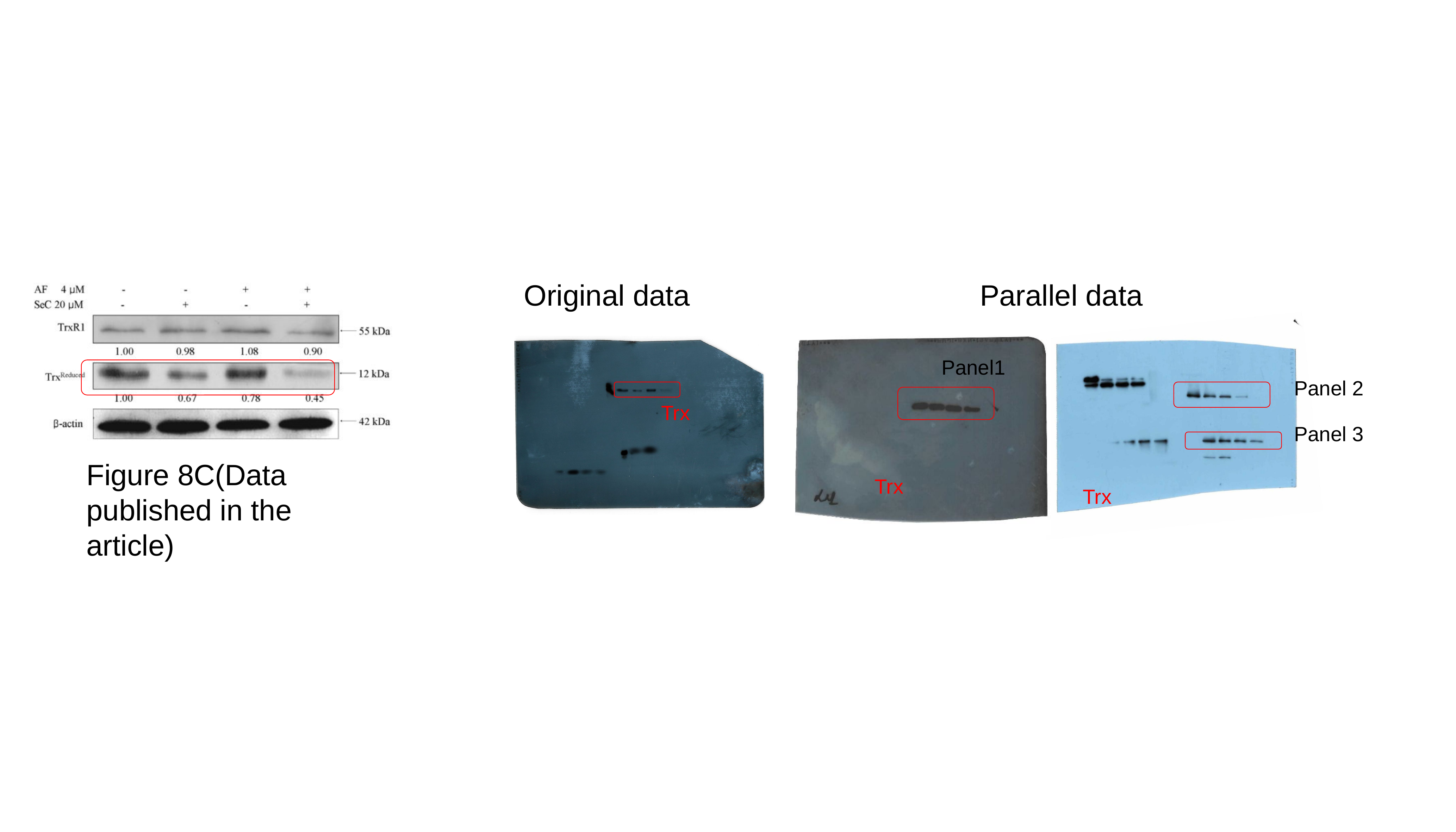

Original data
Parallel data
Panel1
Panel 2
Trx
Panel 3
Figure 8C(Data published in the article)
Trx
Trx

## Slide 4
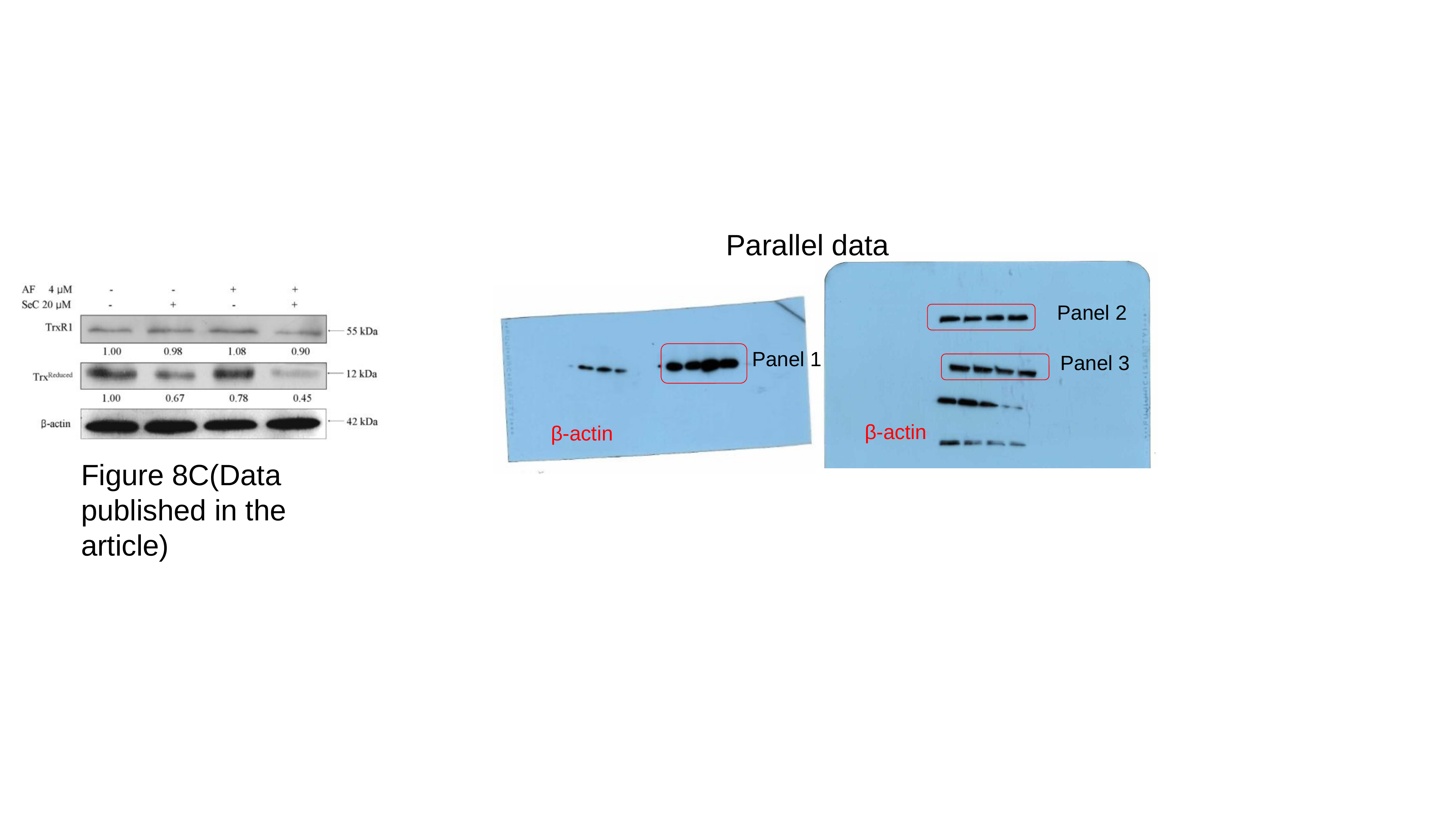

Parallel data
Panel 2
Panel 1
Panel 3
β-actin
β-actin
Figure 8C(Data published in the article)

## Slide 5
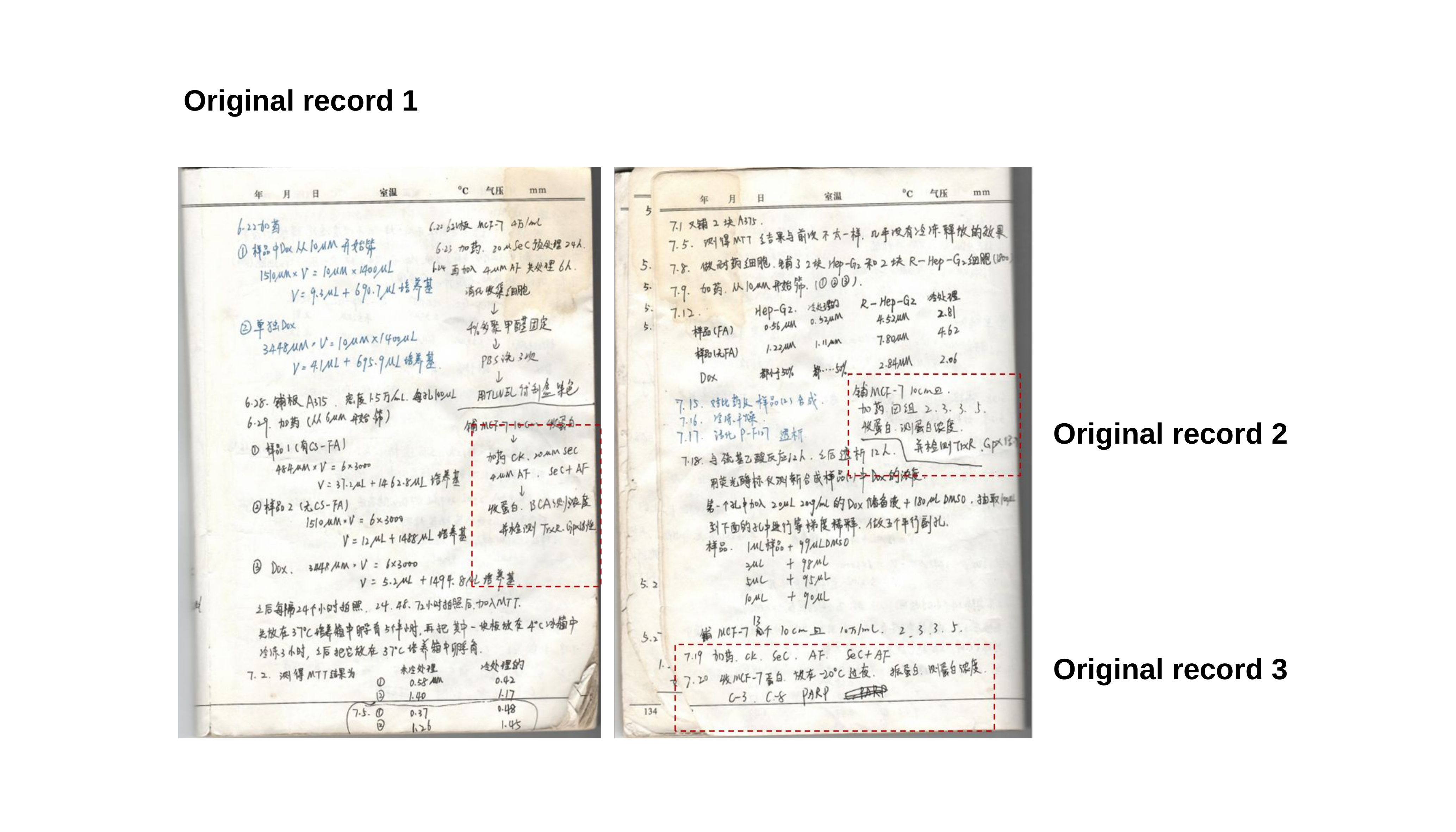

Original record 1
Original record 2
Original record 3
